# Expansion of extrafollicular B and T cell subsets in childhood-onset systemic lupus erythematosus

**DOI:** 10.3389/fimmu.2023.1208282

**Published:** 2023-10-27

**Authors:** Ryan M. Baxter, Christine S. Wang, Josselyn E. Garcia-Perez, Daniel S. Kong, Brianne M. Coleman, Valentyna Larchenko, Ronald P. Schuyler, Conner Jackson, Tusharkanti Ghosh, Pratyaydipta Rudra, Debdas Paul, Manfred Claassen, Rosemary Rochford, John C. Cambier, Debashis Ghosh, Jennifer C. Cooper, Mia J. Smith, Elena W. Y. Hsieh

**Affiliations:** ^1^ Department of Immunology and Microbiology, School of Medicine, University of Colorado, Aurora, CO, United States; ^2^ Department of Pediatrics, Section of Rheumatology, School of Medicine, University of Colorado, Children’s Hospital Colorado, Aurora, CO, United States; ^3^ Center for Innovative Design and Analysis, School of Public Health, University of Colorado, Aurora, CO, United States; ^4^ Department of Biostatistics and Informatics, School of Public Health, University of Colorado, Aurora, CO, United States; ^5^ Department of Statistics, Oklahoma State University, Stillwater, OK, United States; ^6^ Clinical Bioinformatics & Machine Learning in Translational Single-Cell Biology, University of Tuebingen, Tuebingen, Germany; ^7^ Department of Pediatrics, Barbara Davis Center for Diabetes, School of Medicine, University of Colorado, Aurora, CO, United States; ^8^ Department of Pediatrics, Section of Allergy and Immunology, School of Medicine, University of Colorado, Children’s Hospital Colorado, Aurora, CO, United States

**Keywords:** SLE, nephritis, CyTOF, interferon, anergic B cells, T peripheral helper cells, plasmablasts

## Abstract

**Introduction:**

Most childhood-onset SLE patients (cSLE) develop lupus nephritis (cLN), but only a small proportion achieve complete response to current therapies. The prognosis of children with LN and end-stage renal disease is particularly dire. Mortality rates within the first five years of renal replacement therapy may reach 22%. Thus, there is urgent need to decipher and target immune mechanisms that drive cLN. Despite the clear role of autoantibody production in SLE, targeted B cell therapies such as rituximab (anti-CD20) and belimumab (anti-BAFF) have shown only modest efficacy in cLN. While many studies have linked dysregulation of germinal center formation to SLE pathogenesis, other work supports a role for extrafollicular B cell activation in generation of pathogenic antibody secreting cells. However, whether extrafollicular B cell subsets and their T cell collaborators play a role in specific organ involvement in cLN and/or track with disease activity remains unknown.

**Methods:**

We analyzed high-dimensional mass cytometry and gene expression data from 24 treatment naïve cSLE patients at the time of diagnosis and longitudinally, applying novel computational tools to identify abnormalities associated with clinical manifestations (cLN) and disease activity (SLEDAI).

**Results:**

cSLE patients have an extrafollicular B cell expansion signature, with increased frequency of i) DN2, ii) Bnd2, iii) plasmablasts, and iv) peripheral T helper cells. Most importantly, we discovered that this extrafollicular signature correlates with disease activity in cLN, supporting extrafollicular T/B interactions as a mechanism underlying pediatric renal pathogenesis.

**Discussion:**

This study integrates established and emerging themes of extrafollicular B cell involvement in SLE by providing evidence for extrafollicular B and peripheral T helper cell expansion, along with elevated type 1 IFN activation, in a homogeneous cohort of treatment-naïve cSLE patients, a point at which they should display the most extreme state of their immune dysregulation.

## 1 Introduction

Systemic lupus erythematosus (SLE) is a systemic multi-organ autoimmune disease with heterogeneous clinical presentation and an unpredictable waxing/waning disease course. Both features pose significant management challenges, as current diagnostic measures are insufficient to predict; i) severity of current/future organ involvement, ii) disease flare, and iii) response to specific immunomodulatory therapy. Childhood onset SLE (cSLE) poses additional challenges as these patients present with worse disease, including higher prevalence of central nervous system and renal involvement (lupus nephritis, LN), with consequent increased morbidity and mortality compared to adult-onset patients (1–3). Up to 80% of cSLE patients develop lupus nephritis (cLN), compared with 50% of aSLE (aLN). Prognosis is poorer in cLN with up to 75% of cLN classified as Class III or IV disease (3, 4). In LN, failure to achieve renal remission at 6 and 12 months after initiation of therapy is associated with end stage renal disease (ESRD)/dialysis (5). This is particularly concerning for children who, by definition, have a longer lifetime to accrue organ damage. Despite this greater risk of disease development and progression, eligibility for LN clinical trials is frequently restricted to patients >18 years, limiting generalizability of published clinical data to pediatric patients.

Despite improved renal outcomes in proliferative lupus nephritis following standardization of cyclophosphamide and mycophenolate mofetil (MMF) treatment regimens, up to 45% of these patients do not achieve remission within the first 6 months of standard therapy (6). In addition, protocolized repeated kidney biopsies have shown continued histologic disease activity in a significant portion of patients achieving apparent complete clinical remission (7). In recent years, lupus nephritis clinical trials have shown limited success, including large, randomized control trials demonstrating no benefit of drugs targeting diverse immune mechanisms, such as co-stimulatory blockade (CTLA4-Ig, abatacept) (8, 9), B cell depletion (anti-CD20, rituximab (10, 11) and ocrelizumab (12)), and cytokine blockade (anti-IL-6, sirukumab (13)). The i) poor rate of clinical remission, ii) limited clinical trial data in pediatric populations, and iii) lack of sensitivity and specificity of conventional histologic renal biopsy classifications to predict response to therapy (14–16), emphasize the urgent need to decipher underlying immune mechanisms driving cLN in order to predict response to currently-approved and novel-targeted therapies.

While it is well established that a breakdown in tolerance of both autoreactive T and B cells contributes to the pathogenesis of SLE, knowledge of the role of specific lymphocyte subset derangements in different ‘SLE phenotypes’ (i.e., organ-specific involvement) remains unknown. Despite the clear role of autoantibody production in SLE, targeted B cell therapies such as rituximab (anti-CD20) and belimumab (anti-BAFF) have shown only modest efficacy (10–12), particularly in cLN. While many studies have linked dysregulated germinal center formation to SLE pathogenesis, other work supports a role for extrafollicular (EF) B cell activation in generation of cells secreting pathogenic antibodies (17, 18). However, whether extrafollicular B cell subsets, their cytokine production, and downstream effects on their T cell counterparts play a role in specific organ involvement or track with disease activity remains poorly understood. Autoreactive B cells that escape central tolerance in the bone marrow and populate the periphery are normally tolerized by anergy, a mechanism that renders self-reactive B cells unresponsive to stimulation due to increased activity of inhibitory signaling circuitry. Anergic autoreactive B cells (Bnd), have been identified in healthy humans (19). Type 1 diabetes (T1D) and autoimmune thyroid disease (AITD) are associated with incipient loss of Bnd cells from the peripheral blood suggesting activation of these self-reactive B cells and their instigation of disease (20, 21). Further supporting this concept, a subpopulation of Bnd cells that expresses activation markers (Bnd2, activated and formerly anergic B cells), has been shown to be increased in T1D patients that develop disease at an early age (22).

The antibody secreting cells (ASC) found in peripheral blood within a few weeks following active immunization are primarily CD38^+^CD27^+^ plasmablasts (PB). Increased frequency of PB in blood is also correlated with SLE disease activity (23–25). Likewise, longitudinal whole blood transcriptome profiling of cSLE patients demonstrated increased expression of a PB mRNA module correlated with increased disease activity (26). Generation of ASC occurs canonically in germinal centers with help from PD1^hi^ CXCR5^+^ T follicular helper cells (Tfh), but can also occur through extrafollicular pathways with help from PD1^hi^ CXCR5^-^ T cells (T peripheral helper, Tph) (27). One recent study demonstrated that increased frequency of Tph cells correlated with disease severity in adult SLE patients, underscoring the potential importance of extrafollicular T/B interactions in SLE (28). Moreover, recent studies have highlighted an important role for the extrafollicular B cell response in both adult and pediatric SLE through the generation of CD27^-^IgD^-^ double negative B cells (DN2) that lack expression of CD21 and CXCR5 (29, 30). Because these DN2 cells do not express the chemokine receptor CXCR5, which is important for homing to follicles, this B cell subset is likely to be extrafollicular in localization. In addition, the DN2 compartment has been shown to be enriched in autoreactive B cells that are precursors of autoantibody secreting ASCs (30–33). Taken together, when B cell tolerance mechanisms such as anergy fail, either because of receipt of T cell help or alterations in inhibitory signaling (i.e., Tph, Tfh, or T cell subsets), these B cells can be activated and enticed to participate in disease pathogenesis. Recent studies indicate that SLE development may involve extrafollicular autoimmune responses that result from coordination of Tph and DN2 cells (28, 30). However, such extrafollicular T/B signature has not been studied in cSLE with or without cLN.

However pathogenic ASC arises, immune complexes (IC) containing antibodies they produce can deposit in various tissues leading to inflammation and end-organ damage. Previous studies using single-cell RNA sequencing of dissociated kidney biopsy samples from adult LN patients and healthy controls revealed evidence of activation of B cells with an age-associated B cell (ABC) signature (34). ABC are very reminiscent of DN2 cells (increased gene expression of TBX21 (Tbet), FCRL5, and ITGAX (CD11c) (35, 36). These studies also revealed evidence of progressive monocyte differentiation in the kidney. A clear interferon response was observed in most dissociated renal biopsy cells but was most pronounced in B and CD4^+^ T cells, suggesting a role for these cells at the diseased tissue-level (37). However, such studies have not been pursued in cLN, despite renal involvement occurring in up to 80% of cSLE and being the greatest predictor of mortality (38). Therefore, there is a strong need to understand which cell types i) drive the development of cLN (37, 39–41), and ii) what biomarkers are predictive of cLN development/progression over the course of cSLE disease (42).

While previous studies have shown that different cSLE phenotypes are associated with a unique immune signature (26), such studies have been limited by several factors including cross-sectional analysis, patient’s previous immunosuppressive treatment, and/or use of only one analytic modality. To better guide patient specific therapy in cSLE, and in particular SLE with cLN, we characterized the immunologic status of treatment-naïve cSLE patients with and without cLN both at diagnosis and over time (longitudinal follow up over 1-2 years), compared to age and sex-matched healthy controls. We integrated clinical laboratory and exam, mass cytometry (CyTOF), and gene expression data (DxTerity®). Paired with unbiased computational analyses to capture high-dimensional phenotypic (i.e*.*, cell identity) and functional (i.e*.*, cytokine, activation, interferon-response) parameters, relevant to ‘patient-level’ and ‘organ-level’ phenotypes, we identified biologically meaningful signals that have escaped conventional approaches.

## 2 Results

### 2.1 Demographic and clinical characteristics of cSLE patient cohort

24 cSLE patients and 30 age and sex-matched healthy controls (HC) were enrolled in the study. Participants’ demographics and baseline clinical and laboratory characteristics are summarized in **Table 1**. The age and sex distribution between cSLE and HC cohorts was similar. The racial/ethnic distribution within the cSLE and HC groups was different, with a Hispanic predominance in the cSLE patients (p = 0.01). The average age at study enrollment for the cSLE cohort was 14.6 years (SD = 2.6), with a female predominance (75%). The demographic characteristics of our cSLE patient group reflect the incidence/prevalence of cSLE disease in general, which has a predominance of female patients of minority racial/ethnic background. Information about ancestral genetic backgrounds in this study was limited to self-reporting of demographic information and thus did not allow deeper exploration of genetic influences within our predominantly Hispanic patient group. We also did not collect any specific SES information with which to explore such associations.

**Table 1:** Demographic, Clinical and Laboratory Information.

	Healthy Control	SLE	p-value
	n = 30	n = 24	
Age at enrollment in years, mean (SD)	13.5 (4.3)	14.6 (2.6)	0.23
Female, n (%)	21 (70.0)	18 (75.0)	0.68
Race/Ethnicity, n (%)			0.01
White/Caucasian	13 (43.3)	2 (8.3)	
Hispanic	9 (30.0)	17 (70.8)	
Black or African American	5 (16.7)	3 (12.5)	
Asian or Pacific Islander	3 (10.0)	2 (8.3)	
Clinical and Laboratory Characteristics of the SLE Cohort			
Age at SLE diagnosis in years, mean (SD)		14.2 (2.6)	
Total study visits, mean (min - max)		3 (1 - 7)	
Total follow up time in weeks, median (IQR)		42.7 (11.3 - 79.4)	
SLEDAI-2K at enrollment, median (IQR)		14.5 (8.0 - 18.5)	
Change in SLEDAI-2K^a^, median (IQR)		7.5 (2.0 - 14.3)	
Proliferative Lupus Nephritis, n (%)		7 (29.2)	
ISN-RPS Class			
Class III, n (%)		1/7 (14.3)	
Class IV, n (%)		6/7 (85.7)	
Concurrent Class V, n (%)		4/7 (57.1)	
Baseline eGFR in mL/min/1.73 m^2^, median (IQR)^b^		118.2 (84.6 - 124.7)	
Baseline Proteinuria in mg pr/mg cr, median (IQR)^c^		2.9 (0.9 - 7.2)	
Individuals who reached LLDAS^d^, n (%)		7/20 (35%)	

SD, standard deviation; IQR, interquartile range; SLEDAI-2K= systemic lupus erythematosus disease activity index-2000.

ISN-RPS, International Society of Nephrology-Renal Pathology Society; eGFR, estimated glomerular filtration rate.

a Calculated as baseline SLEDAI – last study visit SLEDAI.

b Modified Schwartz equation.

c Proteinuria assessed via spot urine protein to creatinine ratio.

d Modified lupus low disease activity state defined as SLEDAI 2K ≤4; without major organ activity; no new disease activity; prednisolone ≤7.5 mg/d and standard immunosuppressant dosage.

Almost every patient in our cSLE patient group was enrolled at diagnosis, except for one patient who was enrolled during a flare episode after medication discontinuation for several years prior to flare. At baseline (enrollment), the median SLEDAI score was 14.5 (IQR 8.0 – 18.5), consistent with moderate-to-high disease severity. The most common clinical features were arthritis (66.7%), cutaneous manifestations (54.2%), and cytopenias (54.2%) (**Supplementary Table 1**). Seven patients (29%) had lupus nephritis (cLN) at diagnosis based on kidney biopsy; all cLN patients had Class III or IV proliferative nephritis (International Society of Nephrology and the Renal Pathology Society, ISN/RPS), and 4/7 (57.1%) had concurrent membranous disease (ISN/RPS class V), reflecting typical severity range of cLN (**Table 1**).

The median follow-up duration for the cSLE patients was 42.7 weeks (IQR 11.7 – 79.4) with an average number of three total study visits (min 1 – max 7, **Supplementary Figure 1A**). Study visits were concurrent with routine clinical care follow up, and hence most visits occurred during the first year of diagnosis when follow up is more frequent. All patients received treatment with hydroxychloroquine and 90% received corticosteroids. The most prescribed immunosuppressive steroid sparing agent was mycophenolate mofetil (65%). Other targeted biologics, including rituximab, belimumab and abatacept, were used sparingly with only one individual having received either therapy (**Supplementary Table 2**). Most patients experienced improvement in disease activity as evidenced by a decrease in median SLEDAI score over time (**Supplementary Figures 1A, B**). The median change in SLEDAI from first to last study visit was 7.5 (IQR 2.0 – 14.3); the maximal improvement in SLEDAI score of 20 points, occurred in one patient. 7/24 patients (35%) reached lupus low disease activity state (LLDAS) during the study follow-up period (**Supplementary Figures 1A, B**), consistent with the increased disease severity in the pediatric population.

Of the seven patients with cLN at enrollment, six had longitudinal data, and of these, four achieved a partial renal response, and none achieved a complete response. Definitions for partial and complete response followed previously published metrics (43). Proteinuria and total SLEDAI-2K values improved compared to baseline values (**Supplementary Figures 1B, C**). None of the cLN patients achieved lupus low disease activity state (LLDAS) during the study period, consistent with the high morbidity/mortality of LN (**Supplementary Figure 1A**). One additional patient developed new-onset proliferative LN one year after enrollment (and sample analysis) and experienced a refractory course.

### 2.2 Newly diagnosed cSLE patients demonstrate a type I interferon signature and downstream immune cellular activation

**Figure 1:**
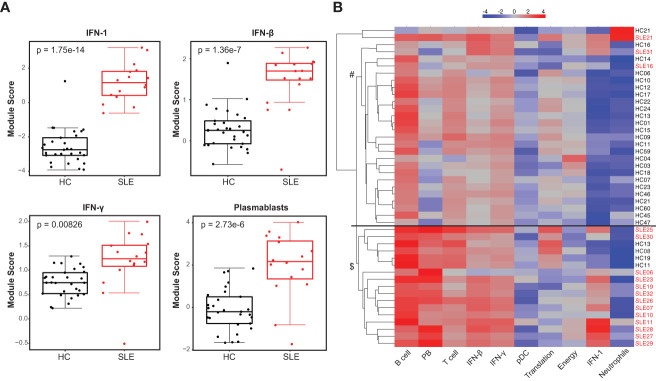
Untreated cSLE patients demonstrate enhanced interferon and plasmablast gene expression signatures compared to HC. HC (black, n=29) and SLE (red, n=16) subjects were assessed for gene expression of 10 mRNA modules. **(A)** Box plots (median, Q1, Q3) of module score (mean of Log_2_ normalized expression of constituent genes). p-values shown within each module comparison determined by Mann-Whitney U test with FDR correction; signficance<0.05. **(B)** Subject-wise dendrogram generated by unsupervised clustering of subjects (rows) according to modular gene expression (columns). Black line at 2^nd^ branch level indicates significant enrichment (p = 4.8e-8) of SLE samples in lower branch ($, SLE n = 13/16, 81%; HC n = 4/29,14%) compared to the upper branch (#, SLE n = 3/16, 19%; HC n = 25/29, 86%). Clustering and testing detailed in methods.

We investigated the whole blood transcriptional profile of cSLE patients using a targeted gene expression panel that assesses 10 gene expression ‘modules’ (i.e., groups of genes) covering different cell types and downstream signaling pathways previously implicated in SLE immunopathogenesis (DxTerity®, see Methods). It is well-established that SLE patients exhibit increased interferon-stimulated gene (ISG) expression at the time of diagnosis, with wide variability in their specific ISG signatures (26). 3/10 DxTerity® gene modules cover ISG targets induced by type I/II IFN ligands, and they all demonstrated statistically significant increased gene expression in cSLE baseline visits versus HC (**Figure 1A**, **Supplementary Figures 2A, B**). Consistent with previous reports of an increased peripheral plasmablast (PB) mRNA signature in pediatric and adult SLE (26, 44), the PB module was significantly elevated in our cSLE cohort (**Figure 1A**, **Supplementary Figure 2C**), while B, T, and dendritic cells (DC), and neutrophil modules showed no significant difference (data not shown).

Unsupervised hierarchical clustering of all baseline cSLE and HC samples based on modular gene expression profiles grouped most cSLE distinctly from HC (p = 4.8e-8, with most cSLE grouped together in bottom half; SLE n = 13/16, 81%; HC n = 4/29, 14%; **Figure 1B**). In addition to these modules, we queried expression of specific gene targets complementary to the mass cytometry analysis, discussed below. Notably, CD38, a marker highly expressed on PB and indicative of T cell activation, was significantly elevated in cSLE compared to HC, along with the lymphocyte activation/exhaustion markers such as PDCD1 (PD-1) and LAG3 (**Supplementary Figures 2C, D**). While all of the individual genes comprising the type I and II IFN, PB, and activation/exhaustion T cell modules were significantly different between cSLE and HC groups (**Figure 1**, **Supplementary Figure 2C**), only PDCD1 and LAG3 were significantly different between cSLE patients with and without LN (**Supplementary Figure 2C**). While not significant, all plasmablast module genes demonstrated an elevated trend in the cSLE with cLN patients when compared to those without cLN, except for BAFF (LN vs. No LN; **Supplementary Figures 2C, D**). These data suggest that B cell activation in particular may play a role in cLN immunopathogenesis.

**Figure 2:**
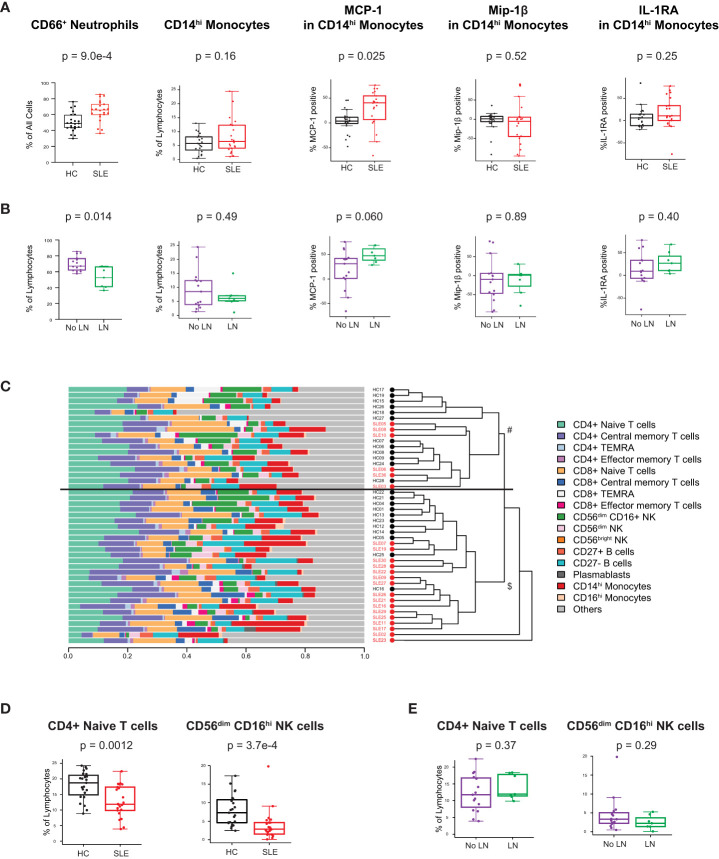
Untreated cSLE patients demonstrate lymphoid and myeloid cellular features consistent with elevated interferon signaling compared to HC. Mass cytometry analysis of HC (black, n=23) and SLE (red, n=22) subjects without LN (purple, n=15) or with LN (green, n=7) at time of diagnosis **(A)** For HC vs. SLE, CD66^+^ Neutrophils frequency from all live cells, CD14^hi^ Monocytes frequency from all lymphocytes, and percent of these cells expressing cytokines (MCP-1, Mip-1 β , IL-1RA) after 6 hours with protein transport inhibitor relative to 95^th^ percentile time-zero threshold (methods). **(B)** Same as A, for No LN (purple) vs. LN (green). **(C)** Compositional Analysis using Kernels (CODAK) of 16 manually gated immune cell subsets (listed with color code at right) shows the proportion of each cell type (x-axis) per subject (y-axis). Overall significance of disease-specific compositional difference, p = 1.2e-4. Row-wise dendrogram generated by unsupervised clustering of subjects according to cell type composition. Black line at 3^rd^ branch level indicates significant enrichment (p = 2.19e-7) of SLE subjects clustering in the lower branch ($, SLE n = 16/22, 73%; HC n = 11/23, 48%) compared to the upper branch (#, SLE n = 6/22, 27%; HC n = 12/23, 52%). **(D, E)** Frequency of populations shown as percentage of lymphocytes (Y-axis), comparing HC vs. SLE **(D)** and SLE patients with LN and No LN **(E)**. For all box plots (median, Q1, Q3) p-values shown within each module comparison determined by Mann-Whitney U test with FDR correction; signficance<0.05.

We analyzed peripheral leukocytes via mass cytometry to investigate alterations of immune cell subsets and their functional state (Methods, **Supplementary Table 3**). 26 surface markers delineated lymphoid and myeloid cell subsets including T, B, NK, DC, monocytes, granulocytes, and their cellular activation states (gating of representative mass cytometry data in **Supplementary Figure 3**). Intracellular proteins measured 14 innate pro-inflammatory and T cell-specific cytokines in response to the *in vivo* ‘SLE inflammatory state’ (no *ex vivo* stimulation, only protein transport inhibitor added). The prominent type I IFN gene expression signature observed in the cSLE cohort at diagnosis (p = 1.75e-14, median score SLE = 1.16 vs. HC = -2.75; **Figure 1A**) is also reflected in increased CD66^+^ neutrophil frequency, and monocyte cytokine production (**Figure 2A**). We have previously identified a pro-inflammatory cytokine signature in CD14^hi^ monocytes of newly diagnosed cSLE patients defined by increased intracellular levels of monocyte chemoattractant protein 1 (MCP-1 or CCL2), macrophage inflammatory protein 1-beta (Mip-1 β or CCL4) and interleukin-1 receptor antagonist (IL-1RA) (45). That same study showed that type I IFN signal is necessary, but not sufficient to induce these cytokines. In the present study we observed a statistically significant increase in the percentage of MCP-1^+^ CD14^hi^ cells (p = 0.025, median SLE = 40.4% vs. HC = 3.63%) in cSLE, as well as a trending, though not significant, increase in the percent of IL-1RA^+^ CD14^hi^ cells, compared to HC (**Figure 2A**). We did not observe a difference in the percent of Mip-1 β ^+^ CD14^hi^ cells (**Figure 2A**). Consistent with our prior report, no difference in the frequency of CD14^hi^ monocytes between cSLE and HC accompanied this difference in cytokine expression (**Figure 2A**). cSLE patients with LN at the time of diagnosis exhibited a trend towards a higher frequency of MCP-1^+^ CD14^hi^ cells compared to cSLE patients without LN (p = 0.060, median LN = 47.7% vs. No LN = 31.3%) (**Figure 2B**).

To determine overall cellular compositional differences (i.e., differences in immune cell population distribution) between cSLE (at diagnosis) and HC, we applied CODAK (46), a kernel-based statistical learning method optimized for high-dimensional and low sample size data sets (Methods). In brief, this approach uses kernel distance covariance to test whether cell type composition associates with a predictor (i.e., disease state – cSLE vs. HC). This methodology requires non-overlapping input populations, and therefore, only CD27^-^ (naïve), CD27^+^ (memory), and PB B cells were included in this clustering approach, since DN2, Bnd, Bnd2, isotype switched B cells, and other B cell subpopulations studied below overlap with the CD27^+^ and CD27^-^ B cell subsets and PB populations. For the same reason, T follicular and peripheral helper cells were also not included in this analysis. To assess whether and how the cSLE *vs*. HC subjects segregated based on cellular compositional difference, we applied unsupervised clustering based on each study subject’s cellular compositional distribution, which grouped most cSLE samples distinctly from HC (p = 2.19e-7, with most cSLE grouped together in bottom half; SLE n = 16/22, 73%; HC n = 11/23, 48%; **Figure 2C**). Once cellular compositional differences between cSLE and HC were determined to be statistically significant (p = 1.2e-4, **Figure 2C**), specific cell types that significantly contributed to this overall compositional difference were identified. cSLE patients showed significantly lower frequencies of CD4^+^ T naïve (TN) cells and CD56^dim^ NK cells compared to HC (**Figure 2D**), but these cell type frequencies did not differ between LN and No LN groups (**Figure 2E**).

These data are consistent with previous literature describing prominent type I IFN, type II IFN, and PB transcriptional signatures in cSLE patients. Extending these studies, we demonstrate downstream T and B cellular activation consequences of such transcriptional signatures, including increased frequency of ISG-dependent cytokine production by classical monocytes, increased frequency of neutrophils, and decreased frequency of naïve T cells. Taken together, our gene expression and immune cellular profiling data extend previously published findings and demonstrate the robustness of the technical and analytical platforms.

### 2.3 Newly diagnosed cSLE patients demonstrate an activated/exhausted memory T cell phenotype that is correlated with disease activity in LN patients only

We investigated the frequencies and phenotype of manually-gated CD4^+^ and CD8^+^ T cell subsets using CD27 and CD45RA to define cells as naïve (TN, CD45RA^+^CD27^+^), central memory (TCM, CD45RA^+^CD27^-^), effector memory (TEM, CD45RA^-^CD27^+^), and effector memory RA (TEMRA, CD45RA^-^CD27^-^) within each lineage. CD4^+^ TN cells were less frequent in cSLE vs. HC, but no differences in CD4^+^ TCM, TEM, or TEMRA were found (**Supplementary Figure 4A**). Among cSLE patients at diagnosis, there were no differences in CD4 T cell subset frequencies between LN and No LN (**Supplementary Figure 4A**). The frequencies of CD8^+^ TN, TCM, TEM, and TEMRA cells were also comparable between cSLE patients and HC, but an increase in CD8^+^ TCM was found when comparing LN vs. No LN (p = 0.05, median LN = 4.70% vs. No LN = 2.26%; **Supplementary Figure 4A**).

**Figure 3:**
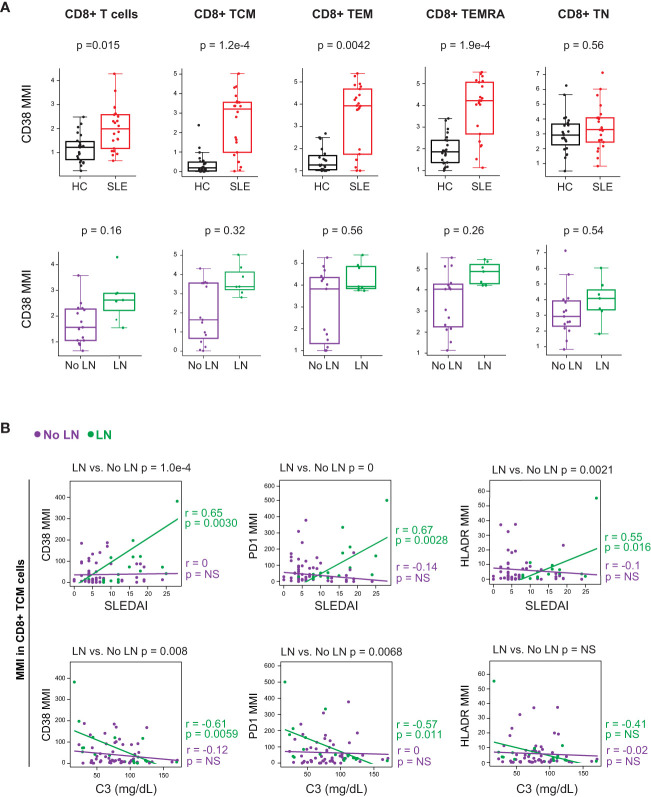
CD8+ T cells from untreated cSLE patients exhibit an activated/exhausted phenotype that correlates with disease score in cLN. Mass cytometry analysis of HC (black, n=23) and SLE (red, n=22) subjects without LN (purple, n=15) or with LN (green, n=7) at time of diagnosis **(A)** or longitudinally **(B)**. **(A)** Median CD38 MMI (arcsinh transformed value) for CD8+ T cell subsets. **(B)** Differential correlations of surface activation marker expression (CD38, PD1, and HLA-DR untransformed MMI) on CD8+ TCM vs. SLEDAI score (left) and C3 (mg/dL; right) for No LN and LN subjects including all timepoints (accounting for repeated measures). p values at top of plots test differential correlations between No LN (purple, n=48) and LN (green, n=16) r and p values at right of plots describe No LN and LN specific correlations. Correlation tests performed by linear mixed model (methods). For all box plots (median, Q1, Q3) p-values shown within each module comparison determined by Mann-Whitney U test with FDR correction; signficance <0.05.

We further investigated these T cell subsets by analyzing their expression of activation markers and cytokines. Non-naïve CD8^+^ T cells from cSLE patients at diagnosis expressed higher levels of the activation markers CD38, PD-1, and HLA-DR (**Figure 3A**, **Supplementary Figure 4B**). However, differences in activation marker expression were not observed when comparing LN *vs*. No LN (**Figure 3A**, **Supplementary Figure 4B**). CD4^+^ TEM cells from cSLE patients expressed more CD38 compared to HC, but no other significant differences in activation marker expression were identified in cSLE vs. HC nor LN vs. No LN (**Supplementary Figure 4C**). We did not find significant differences in T cell IFN- γ , TNF-α, or IL-17 cytokine production between cSLE vs. HC, nor LN vs. No LN, in the absence of exogenous stimulation (only addition of protein transport inhibitor, **Supplementary Figure 4D**).

We applied linear mixed models to test correlations between CD4^+^ and CD8^+^ T cell subset frequencies, and their expression of activation markers, against clinical disease activity metrics over the course of the study (i.e., SLEDAI and complement C3 levels; reduced C3 levels are indicative of increased disease activity). Correlations between T cell activation marker expression and disease activity (SLEDAI) were tested differentially based on LN status at time of diagnosis (**Figure 3B**, differential correlation p-value at top of panels). Expression of CD38, PD-1 and HLA-DR in CD8+ TCM cells correlated with baseline SLEDAI score only in patients with LN, and the corresponding LN-specific inverse correlations to complement C3 levels were observed for CD38 and PD-1 (**Figure 3B**). Expression of activation markers CD38 and HLA-DR was correlated with disease severity in CD8+ T naive (TN) cells but not other CD8+ T cell subsets (**Supplementary Figure 4E**). Additionally, there was no correlation found between the frequency of any CD8^+^ T cell subsets and SLEDAI score, regardless of LN status (**Supplementary Figure 4F**). Overall, these findings support a specific relationship between highly activated CD8^+^ TCM cells and disease severity amongst cSLE patients with LN at diagnosis.

### 2.4 Newly diagnosed cSLE patients demonstrate increased frequencies of circulating T follicular and peripheral helper cells, but the frequency of cTph correlates with disease activity in cLN patients only

**Figure 4:**
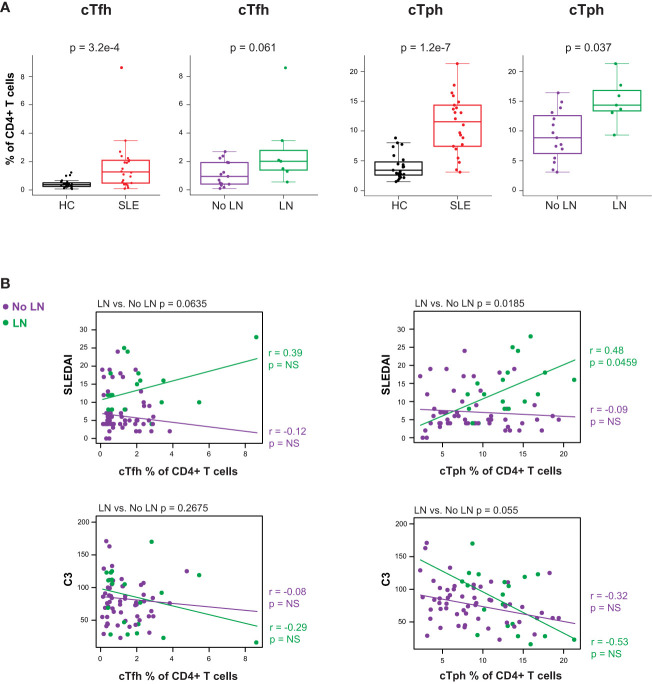
Untreated cSLE patients demonstrate increased frequency of T follicular helper (cTfh) and T peripheral helper (cTph) compared to HC, and cTph frequency correlates with disease activity longitudinally in cLN. Mass cytometry analysis of HC (black, n=23) and SLE (red, n=22) subjects without LN (purple, n=15) or with LN (green, n=7) at time of diagnosis **(A)** or longitudinally **(B)**. **(A)** cTfh and cTph cells as % of CD4+ T cells for HC vs. SLE and No LN vs. LN. **(B)** Differential correlations of cTfh and cTph frequencies at all timepoints vs. SLEDAI score (top) and C3 (mg/dL; bottom) for No LN and LN subjects including all timepoints (accounting for repeated measures). p values at top of plots test differential correlations between No LN (purple, n=48) and LN (green, n=16) r and p values at right of plots describe No LN and LN specific correlations. Correlation tests performed by linear mixed model (methods). For all box plots (median, Q1, Q3) p-values shown within each module comparison determined by Mann-Whitney U test with FDR correction; signficance <0.05.

Considering the prominent role of autoreactive antibody-secreting cells (ASC) in SLE, we evaluated peripheral circulating CD4^+^ T cells known to promote B cell differentiation in either germinal center (cTfh) or extrafollicular sites (cTph) as a surrogate for T-B (and myeloid) cellular interactions. The CD4^+^ T cell compartment of cSLE patients at diagnosis contained higher percentages of both cTfh (PD-1^hi^, CXCR5^+^) and cTph (PD-1^hi^, CXCR5^-^) compared to HC (cTfh: p = 3.2e-4, median SLE = 1.27% vs. HC = 0.371%; cTph: p = 1.2e-7, median SLE = 11.5% vs. HC = 3.43%**;** **Figure 4A**). The frequency of cTph, but not cTfh, was significantly higher in LN patients compared to those without LN at diagnosis (p = 0.037, median LN = 14.3% vs. No LN = 8.87%; **Figure 4A**).

Correlations between cTph or cTfh frequency and disease activity (SLEDAI) were tested differentially based on LN status at time of diagnosis. The frequency of cTph cells correlated with SLEDAI score for LN patients only (LN r = 0.48, p = 0.046; No LN r = -0.09, p = 0.52; differential p = 0.019 at the top of the panel; **Figure 4B**). These data indicate that an increased frequency of cells that promote B cell differentiation into ASC at extrafollicular or inflamed tissue sites is associated with increased disease severity amongst cSLE patients with LN. No such correlation between cTfh frequency and disease activity was observed regardless of LN status (**Figure 4B**), indicating that while cTfh cells are more frequent in cSLE generally, they are not increased in the setting of LN and their frequency does not correlate with disease severity. Taken together, our multi-dimensional analysis of T cells demonstrates; i) a bias toward increased activated CD8^+^ TCM cells coupled with CD4^+^ T helper cells that support B cell differentiation as general features of cSLE, and ii) increased cTph frequency as a correlate of disease activity in LN only.

### 2.5 Newly diagnosed cSLE patients demonstrate increased frequency of extrafollicular B cell subsets

**Figure 5:**
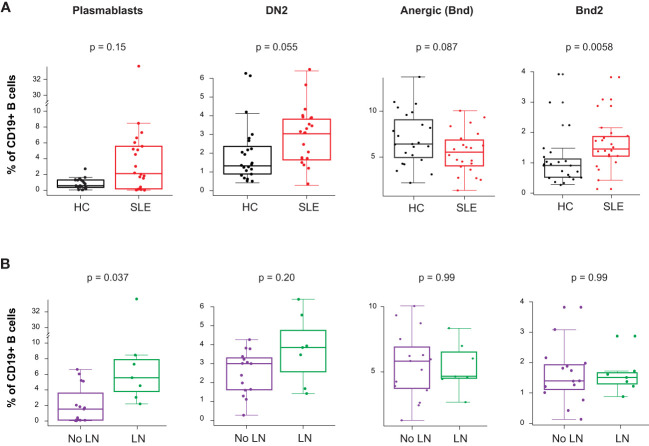
Untreated cSLE patients demonstrate increased frequency of activated B cell subsets compared to HC, and plasmablasts are more frequent in cLN compared to No LN. Mass cytometry analysis of HC (black, n=23) and SLE (red, n=22) subjects without LN (purple, n=15) or with LN (green, n=7) at time of diagnosis. **(A)** Boxplots of B cell subsets shown % of CD19+ B cells in HC vs. SLE. **(B)** Same as A but comparing No LN (purple) vs. LN (green). For all box plots (median, Q1, Q3) p-values shown within each module comparison determined by Mann-Whitney U test with FDR correction; signficance <0.05.

The gene expression analysis showed an increased PB mRNA module score in the blood of newly diagnosed untreated cSLE patients compared to HC (p = 2.7e-6, median SLE score = 2.17 vs. HC = -0.199; **Figure 1A**), and this module trended higher in LN patients (**Supplementary Figure 3C**). The CyTOF analysis demonstrated increased PB frequency (% of total B cells), though this was not significant (p = 0.15, median SLE = 2.12% vs. HC = 0.602%; **Figure 5A**), and the PB frequency was significantly higher in LN patients (p = 0.037, median LN = 5.56% vs. No LN = 1.53%; **Figure 5B**). We evaluated non-plasmablast B cell subtypes defined by CD27^-^ B cell subsets (IgM^-^IgD^-^ atypical memory, IgM^-^IgD^+^ anergic, IgM^+^IgD^-^ immature naïve, IgM^+^IgD^+^ mature naïve) and CD27^+^ B cell subsets (IgM^-^IgD^-^ class switched memory, IgM^-^IgD^+^ c-delta class switched, IgM^+^IgD^-^ IgM memory, IgM^+^IgD^+^ pre-switched) and observed no disease-specific differences in frequency (**Supplementary Figures 5A, B**). However, cSLE patients demonstrated an increased frequency of CD21^lo^ B cells (p = 1.65e-5, median SLE = 42.3% vs. HC = 14.0%; **Supplementary Figure 5B**).

Because it has been previously demonstrated that PB in SLE patients can differentiate from a pool of activated naive cells (aNAV) (47, 48), we evaluated aNAV population frequency as defined by CD27^-^CD38^-^CD21^lo^CXCR5^-^IgD^+^CD11c^+^. We did not find any difference in aNAV frequency between SLE vs. HC, or LN *vs*. No LN (**Supplementary Figure 5A**). aNAV B cells have been shown to be progenitors of DN2 B cells (30, 48). As mentioned above, the DN2 B cell subset can differentiate into ASC via an extrafollicular pathway, and their frequency has been shown to correlate with SLE disease activity (30). In our study, we found this DN2 population to be increased in cSLE vs. HC (p = 0.055, median SLE = 3.04% vs. HC = 1.32%; **Figure 5A**), with an increased trend in LN vs. No LN (p = 0.22, median LN = 3.85% vs. No LN = 3.00%; **Figure 5B**). Because the ASC in SLE likely derive from precursors with autoreactive specificity that escape anergy, we evaluated the anergic B cell compartment (Bnd, CD27^-^IgM^-^IgD^+^) and found a trend toward reduced frequency in cSLE patients (p = 0.087, median SLE = 5.50% vs. HC = 6.40%; **Figure 5A**). This is consistent with observations in other autoimmune diseases that argue a loss of the B cell anergic phenotype occurs in asymptomatic autoantibody positive T1D and new-onset pediatric T1D and AITD patients (21, 49–51). We further evaluated the Bnd compartment and found that Bnd2 cells (CD27^-^IgM^-^IgD^+^CD21^lo^CXCR5^-^), which express increased markers of activation and are thought to be extrafollicular in derivation (52), are increased in cSLE vs. HC (p = 0.0058, median SLE = 1.48% vs. HC = 0.913%; **Figure 5A**). Neither DN2, Bnd, nor Bnd2 cell frequencies were significantly different between LN and No LN groups (**Figure 5B**). These findings demonstrate increased frequency of extrafollicular B cellsin cSLE, consistent with breakdown of the B cell tolerance.

Given the correlations between cTph and SLEDAI disease activity in cLN, we also evaluated whether different B cell subset frequencies correlated with disease activity over the course of disease. While such analyses demonstrated lack of correlation of any of the *a priori* user defined populations with SLEDAI (**Supplementary Figure 5D**), the application of unsupervised machine learning algorithms showed otherwise, as described below.

### 2.6 An extrafollicular B/T cell signature correlates with disease activity in LN patients only

To further explore the differences in the B cell compartment, we leveraged a machine learning tool designed to identify rare and/or heterogeneous disease-associated cell signatures based on many parameters simultaneously. CellCnn is a supervised, neural network-based (Cnn), multiple instance representation learning algorithm that has been used to identify disease-associated cell signatures in another autoimmune disease such as multiple sclerosis (53, 54). In brief, this tool is trained to identify a multi-parametric signature (filter) of cells that differs most distinctly between disease states – it is supervised with respect to disease state but is agnostic with respect to any user-defined definition of cell gates or functional features. The researcher can then query the frequency and characteristics of cells strongly conforming to this signature (quantified by CellCnn score) in all samples, to make inferences about cells relevant to disease.

**Figure 6:**
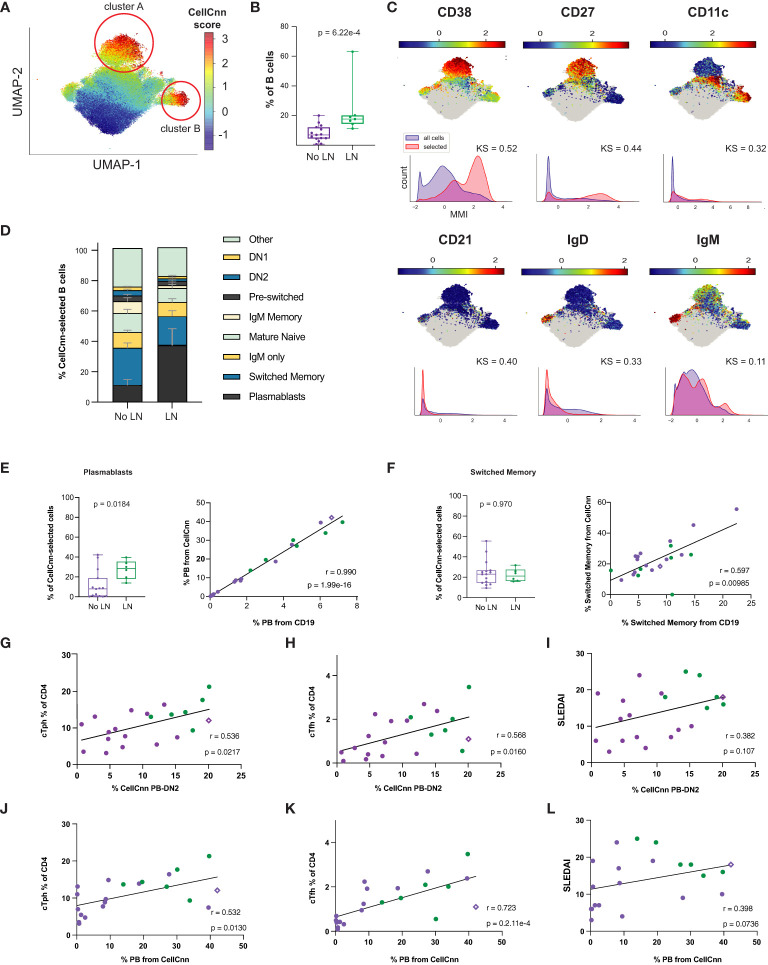
Untreated cLN patients exhibit a B cell disease-specific population spanning plasma cell differentiation that correlates with cTph and cTfh frequencies. Mass cytometry analysis of No LN (purple, n=15) and LN (green, n=7) subjects at time of diagnosis. A supervised neural network-based learning algorithm (CellCnn) was applied to analyze B cells from SLE patients with and without LN at time of diagnosis to identify i) an immunophenotypic signature based on surface markers **(A–C)** that discriminates between No LN and LN. **(A)** UMAP projection composite for all samples showing 85^th^ percentile of cells with CellCnn scores conforming to filter (see methods; CellCnn Score coloration indicates strength of conformity to cell-selection signature). **(B)** Frequency of CellCnn-selected cells in No LN and LN subjects. **(C)** Overlay of relevant characteristic marker intensities on UMAP projection (as in **A**); corresponding histogram of marker intensity between selected (red) and all (blue) B cells (KS = Kolgorov-Smirnov distance). **(D)** B cell subset composition of CellCnn-selected B cells based on manually-gated populations for No LN (n=15) vs. LN (n=6*) groups (methods). **(E)** Boxplots of plasmablast frequency from CellCnn-selected B cells from LN vs. No LN groups. Spearman correlation of PB frequency from % CellCnn-selected B cells vs. manually-gated PB frequency from % total B cells analyzed. **(F)** Same as E but for isotype switched B cells. Spearman correlations of the frequency manually-gated cTph, cTfh cells, and SLEDAI vs. % CellCnn PB-DN2 cells **(G–I)** and those CellCnn-selected B cells that were identical to user-gated plasmablasts **(J–L)**. *For one LN subject, CellCnn-selected cells comprised 98.4% PBs. Subject excluded from panels E-L to calculate correlation coefficient and significance. For all box plots (median, Q1, Q3) p-values shown within each module comparison determined by Mann-Whitney U test with FDR correction; signficance <0.05.

Using surface markers measurements made by CyTOF analysis (**Supplementary Table 3**) as features to select disease-associated cells (those cells that probabilistically and statistically differentiate LN from no LN disease state at diagnosis), we found a heterogeneous subset of B cells that is more frequent in cSLE patients with LN than without LN (p = 6.22e-4, median LN = 17.6% vs. No LN = 6.94%; **Figures 6A, B**). The selected population was comprised of two clusters visualized on a UMAP projection (**Figure 6A**, CellCnn score greater than 3), one with a CD11c^-^CD27^+^CD38^hi^IgD^-^ phenotype (cluster A), and one with a CD11c^+^CD27^-^CD38^mid^IgD^-^ phenotype (cluster B) (**Figure 6C**). These phenotypes resemble PB and DN2 populations respectively, suggesting that CellCnn identifies a continuum of disease-associated B cells along the extrafollicular and ASC differentiation pathway. This continuum most distinctively differentiates LN vs. No LN disease state (at diagnosis). A phenotypically similar CellCnn-associated B cell population (named ‘CellCnn PB-DN2’ for short) was also found to differ significantly between the disease states of cSLE without LN vs. HC (p = 2.56e-7, median SLE = 6.87% vs. HC = 0.725%; **Supplementary Figures 6A, B**).

To further discern the phenotype of the heterogeneous LN-associated ‘CellCnn PB-DN2’ population, these cells were mapped to conventionally gated B cell subsets (on an individual patient basis), and this demonstrated that isotype switched B cells and PB constitute over a third of the ‘CellCnn PB-DN2’ cells that best differentiate LN and No LN status (**Figure 6D**, bottom two populations of the stacked bar graph). However, while the PB frequency from the ‘CellCnn PB-DN2’ is significantly different between LN and No LN (p = 0.018, median LN = 30.11% vs. No LN = 7.84%; **Figure 6E**), the isotype switched B cell frequency (from the ‘CellCnn PB-DN2’) is not (p = 0.97, median LN = 22.92% vs. No LN = 21.21%; **Figure 6F**). The frequencies of both PB and isotype-switched B cells determined from the CD19+ B manual gate (X-axis, % from CD19+, **Figures 6E, F**) correlate with their frequencies determined using the CellCnn selected B cells (Y-axis, % from CellCnn, **Figures 6E, F**). Thus while the abundance of these cells among total CD19+ B cells from each patient is indeed consistent with abundance of cells defined using the CellCnn signature, only the PB frequency is statistically different between LN and No LN. These findings further support a unique role of B cell activation/differentiation toward ASC in cLN immunopathogenesis.

Frequency of LN-associated ‘CellCnn PB-DN2’ cells (% ‘CellCnn PB-DN2’ on X-axis of **Figures 6G, H**) strongly correlated with the frequency of manually gated cTph (r = 0.536, p = 0.022; **Figure 6G**) and cTfh cells (r = 0.568, p = 0.016; **Figure 6H**) in cSLE patients. Particularly, for LN patients who demonstrated higher percentage of ‘CellCnn PB-DN2’ B cells, cTph and cTfh, the correlation amongst them was even more robust and significant (green dots in **Figures 6G, H**), supporting the strong relationship between cTph cells and B cell differentiation toward ASC in LN. Further supporting ASC differentiation as the driving feature distinguishing LN and No LN in this ‘CellCnn PB-DN2’ B cell signature, we found a robust correlation between the frequency of PB population from the CellCnn selected B cells (% PB from CellCnn on X-axis of **Figures 6J-L**) to cTph (r = 0.532, p = 0.013; **Figure 6J**) and cTfh (r = 0.723, p = 2.1e-4; **Figure 6K**). Neither the frequency of these ‘CellCnn PB-DN2’ B cells or PB population from the ‘CellCnn PB-DN2’ significantly correlated with disease activity (SLEDAI) in cSLE (all purple and green dots, r = 0.382, p = 0.11; **Figures 6I, L**). However, while all cLN patients (green dots only) demonstrated the highest frequency of ‘CellCnn PB-DN2’ B cells correlated with the highest SLEDAI scores (**Figure 6I**), they did not demonstrate the same pattern for the percent PB from CellCnn selected cells (**Figure 6L**), suggesting that other B cell populations in the ASC trajectory (extrafollicular and germinal center derived) contribute to LN pathogenesis.

We also investigated T cell and monocyte compartments using the CellCnn methodology but did not detect statistically significant differences associated with LN (or cSLE in general) disease state (data not shown). Overall, our findings using both manual gating and CellCnn suggest an important role for the continuum of extrafollicular T/B interactions toward ASC and their potential involvement in development of LN. Remarkably, one non-LN cSLE patient who at the time of sampling demonstrated an increased percentage of ‘CellCnn PB-DN2’ B cells (and manually gated PB), cTph, and cTfh cells (purple diamond in **Figures 6G-L**), with the same “signature/pattern” as the LN patients (green dots, **Figures 6G-L**), developed LN a year later. These data support the notion that increases in these populations may precede LN development.

### 2.7 A cytokine-producing heterogeneous LN-associated B cell signature is correlated with disease activity in LN patients only

**Figure 7:**
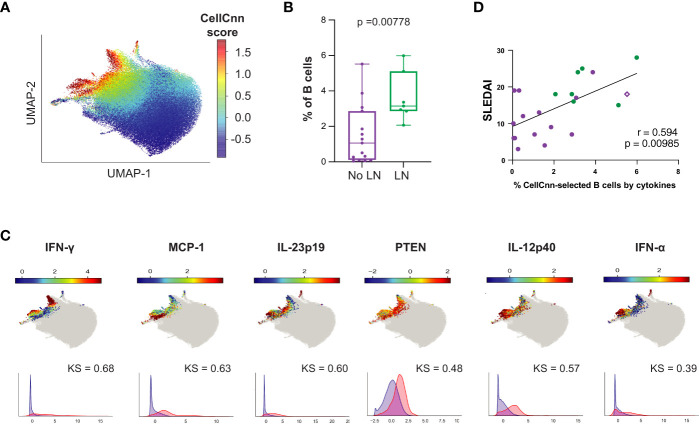
The B cell compartment of untreated cLN patients demonstrates disease-specific signature populations defined by cytokine production that correlates with disease activity. Mass cytometry analysis of No LN (purple, n=15) and LN (green, n=7) subjects at time of diagnosis. A supervised neural network-based learning algorithm (CellCnn) was applied to analyze B cells from SLE patients with and without LN at time of diagnosis to identify signature population based on intracellular cytokine production that discriminates between No LN and LN. **(A)** UMAP projection composite for all samples showing 85^th^ percentile of cells with CellCnn scores conforming to filter (see methods; CellCnn Score coloration indicates strength of conformity to cell-selection signature. **(B)** Frequency of selected cells in No LN and LN subjects. **(C)** Overlay of relevant characteristic marker intensities on UMAP projection (as in **A**); corresponding histogram of marker intensity between selected (red) and all (blue) B cells (KS = Kolgorov-Smirnov distance). **(D)** Spearmann correlation of SLEDAI vs. % of CellCnn-selected B cells by cytokine signature; significance <0.05.

While production of autoantibodies is typically considered the most pathogenic effector function of B cells in SLE, B cells can also act as antigen presenting cells and make pro-inflammatory cytokines, such as IFN- γ , TNF- α , IL-6, IL-23, and IL-12. These functions may influence T cell differentiation and promote Th1-dependent inflammatory processes (55–57). We applied CellCnn analyses to B cells from LN vs. No LN patients based on intracellular cytokine production and identified an LN-associated B cell population (p = 0.00778, median LN = 3.15% vs. No LN = 1.06%; **Figures 7A, B**) with elevated IFN- γ , MCP-1, IL-12p40, PTEN, IL-23p19, IFN- α , and IL-6 (**Figure 7C**). The observed elevated levels of PTEN are likely indicative of recent activation (50). Interestingly, we noted that MCP-1 expression is highly statistically significant in the disease-associated B cells (**Figure 7C**, KS value). MCP-1 was also significantly produced by CD14^hi^ monocytes from LN patients (**Figure 2B**). This disease-associated B cell population was also found to be significantly more frequent in cSLE without LN compared to HC (p = 1.78e-3, median SLE = 2.41% vs. HC = 1.32%; **Supplementary Figures 6C, D**), suggesting the pervasive nature of cytokine producing B cells in cSLE.

The frequency of B cells fitting this cytokine signature was strongly correlated to clinical disease activity at baseline (SLEDAI) (r = 0.594, p = 0.00358, **Figure 7D**); however, the frequency of these cytokine producing B cells did not correlate with cTph or cTfh frequency as the CellCnn surface-marker-defined B cells did (**Supplementary Figures 6E, F**). Interestingly, the subject highlighted in **Figure 6** (purple diamond), who did not have LN at the time of analysis but developed LN approximately one year later, also showed a higher frequency of these cytokine-producing B cells (% CellCnn-selected B cells by cytokines on X-axis) correlated with SLEDAI score (purple diamond, **Figure 7D**). These findings suggest that while the predominant role of B cells in SLE pathogenesis is autoantibody production, a small population of proinflammatory cytokine-producing B cells could have an additional role in LN pathogenesis.

## 3 Discussion

We applied a multi-modal peripheral blood immune profiling approach to cSLE patients, sampling both at diagnosis prior to treatment and longitudinally as their disease was treated/progressed, to identify abnormalities associated with clinical manifestations and disease activity, specifically, LN. Despite the modest size of our study cohort, our findings confirmed themes of previously reported cSLE literature, such as the predominance of an elevated type I IFN and PB signatures, increased neutrophil frequency, increased monocyte cytokine production, and T cell activation/exhaustion. These findings demonstrate that our study cohort represents “typical cSLE pathology” and supports the robustness of our technical platforms. Importantly, we extended knowledge regarding the immunopathogenesis of cLN by identifying i) an extrafollicular T/B cellular (cTph, plasmablast, DN2, Bnd2), and ii) a B cell cytokine-producing signature in cSLE patients that is correlated with disease activity in cLN, suggesting a specific pathway that could be targeted in renal disease. While previous studies have addressed these cell types, their cytokine production repertoire, and different blood transcriptomic profiles separately, our approach integrated these analyses in the same patients. These findings serve to define the specific pathways of B cell differentiation/activation that should be targeted in LN specifically, particularly since general anti-CD20 and anti-BAFF therapies have had mixed therapeutic success.

Unlike other studies where the patient population studied was heterogeneous in clinical disease phenotype and stage/course of disease, our cSLE patient cohort is homogeneous as all patients were studied at diagnosis, when treatment-naïve, a point at which they should display the most extreme state of their immune dysregulation. They were then followed longitudinally for 1-2 years, allowing study of progression of disease phenotype across time. During this interval subjects underwent standard of care treatment and demonstrated changing disease scores (**Table 1**, **Supplementary Tables 1**, **2**, **Supplementary Figure 1**). Consistent with previous literature, we demonstrated that cSLE patients’ peripheral blood demonstrate an enhanced type I and II IFN gene expression signature (**Figure 1**). In newly diagnosed untreated cSLE patients, we evaluated three different interferon-based gene expression modules including DxTerity® IFN-1, IFN- β , IFN- γ , all of which demonstrated statistically significant increases compared to healthy controls. The commercially available DxTerity® IFN-1 module, composed of Herc5, IFI27, IFIT1, and RSAD2, demonstrates the greatest significance in cSLE vs. HC. This gene expression module has been previously shown to be a key prognostic marker for SLE patients, as patients with a high baseline IFN-1 score have been shown to have 3X increased risk of developing LN (58). Our findings support the use of this commercially available IFN-1 assay for patient care and clinical trials (59–61). Future studies using a micro-collection device to assess IFN-1 score via fingerprick blood droplet will allow the measurement of type I IFN scores in a consistent longitudinal fashion in large cSLE study cohorts, including cLN specifically. Such validation studies will provide the foundation for a “home based” disease activity monitoring capability and provide data to study predictors of flare.

Type I and II IFN possess pleiotropic downstream effects on T and B cell activation, such as promotion of T cell differentiation (effector and central memory), B cell differentiation into DN2, Bnd2, and ASC, and depending on chronicity of disease process, T and B cell exhaustion (62–66). These IFN downstream immunological consequences were all identified in our studies, including increased frequency of i) extrafollicular (EF) B cell subsets DN2, Bnd2, and PB cells; ii) cTph and cTfh, and iii) CD8^+^ TCM, and expression of CD38, PD-1 and LAG-3 in CD8^+^ T cells (67, 68) (**Figures 2**, **3**). While these findings have been previously addressed in other studies, they have all focused on adult patient cohorts, or incompletely studied – i.e., the studies focused on EF B cell subsets only, or T cell activation only, etc.

Previous studies have demonstrated increased frequency of certain B cell subsets in SLE patients, particularly those with LN. Sanz et al. demonstrated increased PB and DN2 populations in SLE patients with nephritis (30, 69, 70). However, these have not been evaluated in pediatric cohorts. We evaluated B cell subsets in our cSLE patient cohort, and identified increased frequencies of PB in LN vs. No LN patients, and extrafollicular DN2 and Bnd2 cells in cSLE vs. HC, without significant differences in aNAV cells (**Figure 5**). It is notable that Bnd2 cells are likely a subset of the aNAV phenotype but are restricted to IgM-lo/negative Bnd cells with an activated phenotype (19, 50, 71). While these user defined B cell subsets were evaluated, the application of machine learning algorithms to the data demonstrated the novel robust relationship between cTph, extrafollicular B cells, and disease severity in LN (**Figure 6**). We identified a heterogeneous population of LN-associated B cells comprising surface marker phenotypes resembling PB (CD27^hi^CD38^hi^) and DN2 (CD11c^+^CD27^-^IgD^-^) (30, 72) (**Figure 6**). As these machine learning-defined cells did not clearly conform to manually gated definitions, we expect that this set also includes transitional states that would occur as atypical memory cells differentiate toward pathogenic ASC; a fate that has been hypothesized in other reports on such B cells in SLE (30, 72). A strength of this approach is the ability to explore features of a heterogeneous set of disease-associated cells, encapsulating B cells at different stages of ASC differentiation trajectory, whether it be following germinal center or extrafollicular pathways, both of which likely have important roles in SLE pathogenesis. Importantly, frequency of these disease-associated B cells correlated robustly with cTph and cTfh, but only in cLN. Integrating these cellular subsets within the same patients (as opposed to separate cross-sectional studies), correlated with clinical disease scores. These findings reveal the specific immune profiles of cells that are the most important to target using new therapies. Clinical trial outcomes may be improved by refining patient suitability criteria based on these modalities.

LN-associated B cells showed elevated levels of cytokines that have been implicated in promoting T cell activation and differentiation into central memory phenotype (MCP-1), and differentiation of CD4^+^ cells into Tph cells (IL-12p40, IL-23p19, IFN- α ) (27). Consistent with such findings were the increased CD8^+^ TCM, cTph and cTfh populations in cSLE patients, particularly those with LN (**Figures 3**, **4**). We discovered a strong correlation between the frequency of these cytokine-expressing B cells and disease activity score across all SLE patients in our cohort, and all eight nephritis patients had greater than 2% of their total B cells fitting this profile, while only 3/14 non-nephritis cSLE patients exceeded that frequency threshold (**Figure 7**). These findings highlight a previously under-recognized, yet potentially important, role for B cell cytokine production in cSLE pathogenesis, particularly in LN. In addition, our findings support the notion of using PB and extrafollicular B cell subsets in disease monitoring (72) and extends the signature through use of a deep learning-derived profile to capture a complex set of B cells associated with disease.

It has been shown that naïve B cells can be driven to differentiate into the extrafollicular DN2 and Bnd2 cells and further into ASCs, in an IL-21-dependent manner (30, 52, 72). cTfh and cTph cells are producers of IL-21, and it has been shown that Tph cells can drive B cell differentiation into PB in the setting of SLE (27, 28, 73). Complementary to our finding of an extrafollicular B cell signature in LN, we found increased cTph cells in cSLE patients at the time of diagnosis. While we defined cTph and cTfh based on CD4 T cell expression of PD-1 and CXCR5, other markers such as CXCR3 and additional transcriptional factors such as Bcl6 for cTfh were not used and may provide further specificity to the data analyzed. Moreover, cTph frequency correlated with disease activity score (SLEDAI) specifically for patients with LN throughout the study period (**Figure 4**). cTph frequency also correlated with the frequency of surface-marker selected B cells (described above) for all patients at the time of diagnosis, with LN patients exhibiting the highest levels for both cell populations. The increased frequency of cTph cells in the circulation, along with the presence of disease-associated B cells that i) resemble PB and extrafollicular DN2 cells, and ii) produce cytokines implicated in driving Tph differentiation, suggests the possibility for a feed-forward dysregulation in which B cells may drive Tph through cytokine production; and Tph cells, in turn, drive the differentiation of DN2-like B cells into pathogenic ASCs by IL-21 production. This implicates signaling in these cell types as attractive therapeutic targets that would avoid the drawbacks of cell-depleting approaches and potentially afford more precise disease management. Our finding of a cytokine production signature amongst disease-associated B cells suggests that these as markers could be used to monitor disease activity, and as direct drug targets themselves. Future flow cytometry and/or gene expression-based studies evaluating extrafollicular T/B cell subsets and B cell cytokine production in a larger cohort of cSLE patients with and without nephritis holds the potential to inform new therapies for organ-specific disease. It is important to consider the role that elevated and sustained type 1 IFN signaling could play in the model of pathologic T/B cell interactions our data support. Type 1 IFN signaling has been shown to promote the differentiation of autoreactive, T-dependent, extrafollicular plasmablasts in a mouse model of SLE (74), and *in vitro* studies of human T cells demonstrate that IFN- α stimulation can induce CD4 T cell phenotypes consistent with Tph/Tfh differentiation (75). Monoclonal therapy directed against the type I IFN receptor subunit 1 (Anifrolumab, Saphelo) is currently FDA approved for treatment of moderate to severe SLE in adult patients, after a decade without the introduction of any new therapies (76–78). The initial clinical trials in 2020 included SLE patients of diverse clinical presentations, but the more recent 2022 clinical trial focused on treatment of active LN. While the primary endpoint of change in baseline 24-hour urine protein–creatinine ratio (UPCR) was not met, the anifrolumab arm did result in improved numerical renal outcomes, suggesting that disruption of type I IFN signaling can result in improvement of renal disease (79). Understanding the role of this intervention with respect to the aberrant T/B cell interactions in cLN that we and other groups have reported on will be critical to defining the downstream immunopathogeneses of elevated type 1 IFN on SLE generally, as well as any treatment (and perhaps even preventive) value it may have against the development of nephritis specifically. The current possibility to study extrafollicular T/B cell interactions before and after type 1 IFN signaling blockade, particularly through spatial analysis of immune cells in the renal tissue, presents an important opportunity to elucidate the immune pathways underlying cLN.

## 4 Methods

### 4.1 Experimental subject details

24 patients with cSLE were enrolled and consented at Children’s Hospital Colorado Rheumatology clinics between April 2016-2019 and followed longitudinally with routine clinical care and peripheral blood collection until study completion, under an IRB approved protocol for which Dr. Hsieh is the PI. At enrollment (baseline), participants were required to be< 21 years of age, meet the 1997 American College of Rheumatology revised SLE classification criteria (80) (**Table 1**, **Supplementary Table 1**), and be either treatment naïve (new diagnosis) or have ceased treatment for longer than six months. Clinical data including physician exam findings and laboratory data were obtained at each study visit. Medications were prescribed according to standard clinical practice at the discretion of the treating physician (**Supplementary Table 2**). An additional 30 healthy subjects were recruited to serve as cross-sectional age and sex-matched controls (**Table 1**).

#### 4.1.1 Disease activity

To assess disease activity, the Systemic Lupus Erythematosus Disease Index Activity Index 2000 (SLEDAI-2K) (81) was reported at baseline and at each follow-up visit by the treating clinician (**Supplementary Figure 1**, **Supplementary Table 2**). Conventional laboratory parameters were collected with each visit, including but not limited to, complete blood cell count and differential, C-reactive protein, sedimentation rate, serum creatinine, complement components 3 and 4, double-stranded DNA (dsDNA) titers, urinalysis with microscopy and urine protein to creatinine ratio (UPCR) (**Supplementary Figure 1**, **Supplementary Table 1**, **Table 1**).

#### 4.1.2 Lupus low disease activity state

The LLDAS definition was modified from the consensus-based definition published by Franklyn et al (82). A patient was deemed to be in LLDAS if their SLEDAI-2K was ≤4, they had no new disease activity or organ involvement compared with the previous assessment, and the prednisone (or equivalent) dose was ≤7.5 mg daily, on stable maintenance doses of immunosuppressive drugs and approved biological agents. The physician global assessment component was omitted from our definition as this was not routinely performed with clinical care. Summary of SLEDAI and LLDAS status can be found in **Supplementary Figure 1**.

#### 4.1.3 Renal response

Definitions for complete and partial renal response were adapted from the Childhood Arthritis and Rheumatology Research Alliance Consensus Treatment Plan for new–onset proliferative lupus nephritis (43) definitions for substantial and moderate renal response, respectively. Core renal parameters include proteinuria (UPCR), renal function (creatinine clearance), and urine sediment (urine WBCs, RBCs, and casts). Complete renal response was defined as normal estimated glomerular filtration rate (eGFR) by modified Schwartz equation for age (eGFR > 90 mL/min/1.72 m^2^), inactive urinary sediment (<5 WBCs/hpf,<5 RBCs/hpf, and no urinary casts), and UPCR< 0.2. Partial renal response was defined as at least 50% improvement in two core renal parameters without worsening of the remaining renal parameter if eGFR was abnormal at baseline and a maximum UPCR ≤ 1. Non-responders did not fulfill criteria for either complete or partial renal response. Summary of renal response for the patients with lupus nephritis can be found in **Supplementary Figure 1**, **Supplementary Table 1**.

### 4.2 Method details

#### 4.2.1 Blood processing and mass cytometry analysis

Whole blood was collected into heparinized vacutainers. Freshly drawn whole blood was treated with protein transport inhibitor (ebioscience 00-4980-03) in the absence of immune-stimulatory agents, incubated for 6 hours, then lysed and fixed (BD lyse/fix buffer #558049) to remove RBCs. Fixed cells were stored in cell staining buffer (MaxPar Cell Staining Buffer, Fluidigm, # 201068) at -80°C. When specimen numbers for batch processing had been obtained, cells were thawed for downstream CyTOF barcoding and staining steps. Mass tag cell barcoding of fixed samples, followed by antibody staining and permeabilization was performed as previously described (45, 83). Antibody panel detailed in **Supplementary Table 3**.

#### 4.2.2 Targeted gene expression panel analysis

Gene expression analyses of specified modules were measured by DxTerity Diagnostics, Rancho Dominguez, CA USA, using the DxTerity Modular Immune Profile (MIP) test, a chemical ligation-dependent probe amplification and gene expression test with relative quantitative analysis by capillary electrophoresis (84). Modules analyzed were: DxTerity IFN Module (IFN-1), B Cell Module, Energy Module, IFN Beta Module, IFN Gamma Module, mRNA Translation Module, Neutrophil Module, pDC Module, Plasmablasts Module, T Cell Module. Sample testing and analysis was performed directly on PAXgene RNA Stabilized Blood as described by Kim et al. (84). The DxTerity MIP test measures the RNA expression levels of 51 immune response genes relative to the expression levels of 3 housekeeping normalizer genes (ACTB, GAPDH and TFRC). Normalized expression values of each respective response gene were calculated per the following function: Normalized Expression_Gene i -_= Log_2_(Height_Gene i_) – Mean (Log_2_(Normalized Gene Height). A 4-gene Type 1 Interferon (IFN-1) signature score was calculated by averaging the normalized expression values of HERC5, IFI27, IFIT1, and RSAD2 in the MIP panel. The IFN-1 signature score cutoff of -0.5 between IFN high and low was determined based on measurement of 281 healthy human blood samples and placing the cut-off at 2 standard deviations (95^th^ percentile) above the mean healthy IFN-1 score (-0.5). This cut-off falls within the trough of the observed bimodal distribution of IFN-1 scores for this and other cohorts of SLE samples.

### 4.3 Computational and statistical analysis

#### 4.3.1 Box plot and clustering analyses of mRNA data

Mann–Whitney U two-sided tests (85) were used to determine the statistical significance of gene expression between HC and SLE groups (**Figure 1A**). P-values were adjusted using Benjamini-Hochberg false discovery rate and adjusted P-values<0.05 were considered statistically significant. No data were excluded from the analyses.

For validation of subject-wise clustering (e.g., HC subjects are mostly clustered together) or identification of potential batch effects (e.g., a couple of SLE subjects [SLE21 and SLE16] share similarities with HC subjects: **Figure 1B** heatmap), unsupervised hierarchical clustering was performed. Initially, each subject is assigned to its own cluster and then the algorithm proceeds iteratively, at each stage joining the two most similar clusters, continuing until there is just a single cluster. At each stage distances between clusters are recomputed by the Lance-Williams dissimilarity update according to the particular clustering method being used. A matrix of subject-to-subject Euclidean distance values was calculated from the input data matrix (as observed from the **Figure 1B** heatmap) of gene modules and subjects. The Ward’s minimum variance algorithm was used for subject-wise clustering (86). Dendrograms are used in subject-wise clustering to help visualize similarities or dissimilarities between subjects.

#### 4.3.2 CyTOF phenotype and function analysis

For all tests, adjustments for multiple testing were conducted using the Benjamini-Hochberg False Discovery Rate (FDR) approach and adjusted p-values were reported (87).

##### 4.3.2.1 Comparison of CD14^hi^ monocytes percent positive cytokine production

We used two-sample t-tests to statistically test the difference between CD14^hi^ monocyte cytokine production after 6 hours of peripheral whole blood incubation with a protein transport inhibitor, between two disease groups (cSLE vs. HC or LN vs. No LN). The threshold median metal intensity (MMI) was determined for each cytokine to be the 95th percentile at T0 for the anchor sample that was included in each barcode set (aliquoted and frozen cells from a single healthy subject to control for technical variability). For each study subject, the percentage of cells expressing a given cytokine after 6 hours was determined relative to this threshold, and the baseline percentage at T0 was subtracted from this. Negative values indicate that for some samples the percentage of above-threshold cells at T6 was less than at T0.

##### 4.3.2.2 Immune cell subsets compositional analysis (cSLE vs. HC) and comparison of frequencies of specific immune cell subsets (HC vs. cSLE and LN vs. No LN)

To analyze the cell-type abundance data, first we obtained the multivariate cell-type compositions of different cell subpopulations with the total lymphocytes as the denominator. These subpopulations were obtained from a hierarchical gating structure using subpopulations that were not expected to have overlap. Cell-type proportions (proportion with respect to the number of lymphocytes) were obtained from this hierarchical tree. This cell-type proportion data was compositional in nature with the proportions summing to 1. We tested the difference in these cell-type compositions between cSLE and HC using a newly developed kernel-based statistical test CODAK (46). Upon finding significantly different cell-type compositions across the two disease groups using CODAK, we followed up using logistic generalized linear mixed models to test for differential abundance of each individual cell-type to identify the most significantly different subpopulations (88). The logistic mixed models were also used to test differential abundance for cell sub-types that were outside the hierarchical tree structure due to overlap with other cell-types. The top two contributors identified included CD4^+^ Naïve T cells and CD56dim CD16^+^ NK. This procedure was repeated for T-cell subpopulations with the T-cells as the denominator. A similar approach using logistic mixed model was used to test the difference in cell-type abundance for the additional individual cell subpopulations: Plasmablasts, DN2, Bnd, and Bnd2 using B-cells as the denominator; cTph and cTfh using CD4^+^ T-cells as the denominator. For the compositional data, we also constructed hierarchical cluster dendrograms of subjects having similar cell-type compositions. Aitchison distance was used as the appropriate distance between two compositions (89).

##### 4.3.2.3 Cellular activation marker expression analysis

Arcsinh transformation with cofactor = 5 was performed on all CyTOF data before analyzing the activation marker expression. Two-sample Wilcoxon-Mann-Whitney tests were used (due to asymmetry in the data) to test the difference in MFI for each activation marker-cell subpopulation combination across the two disease groups (cSLE VS. HC or LN VS. No LN).

##### 4.3.2.4 Correlations of immune cell subsets or cellular activation markers to each other and/or to disease activity scores/metrics

For the longitudinal part of the study, we used data from all time points (cSLE only) to test differential correlation between marker-abundance, marker-disease score, or abundance-disease score pairs across the lupus nephritis disease groups (LN vs No LN). Linear mixed model was used to conduct these tests.

#### 4.3.3 Application of CellCnn algorithm to CyTOF data

For CellCnn analysis, patient samples at time of diagnosis were divided into two disease categories: with LN (n=7) and without LN (n=15). The training and validation sets comprised.fcs files from both categories that were manually pre-gated for CD19+ B cells. The network was trained by randomly initializing the *filters* (weights of the convolution part of the network) from a continuous uniform distribution [-0.05, 0.05]. A filter is a vector having a length equal to the number of cytometry markers considered in the analysis. Markers considered for the surface phenotype analysis, and the cytokine analysis are detailed in **Supplementary Table 3**. The number of filters is a hyperparameter chosen randomly from a range of 3 to 10 and optimized using a random search. Other hyperparameters including learning rate and dropout are optimized following the same strategy.

After performing convolution operation on multi-cell inputs (see Arvaniti, 2017 (53)), a top-k pooling (mean of top-k cells, where k ranges from 0.1, 1, 5, 20, up to 100% of a total number of cells in a batch of 200 cells) strategy is applied. The mean of top-k pooled response evaluates the frequency of a cell subset having top k% of cells ordered according to the CellCnn score obtained from the convolution operation by a particular filter. For each of the initialized filters, we obtain one pooled response. Therefore, the vector of pooled response has the same dimension as the number of initialized filters. Finally, the output layer performs a weighted sum operation over the pooled response and applies a softmax (for classification task) function to obtain the final response of the network. The network is trained and validated using 5-fold cross-validation on multi-cell inputs constructed from samples. During training, the network uses mini-batch (batch size=200) stochastic gradient descent with Adam optimizer and categorical cross-entropy as the loss function to optimize the network weights (90). Upon obtaining the model with the lowest validation loss, corresponding trained filter weights were used to assign each cell a cell-filter response score called the CellCnn score. A suitable percentile threshold (e.g., 85%) was chosen to identify the cells from each sample with a CellCnn score exceeding that threshold. UMAP was then used to visualize cell similarity relationships in 2D with above-threshold CellCnn scores represented as a color gradient overlay (**Figures 6**, **7**). To assess whether individual marker expression on above-threshold cells differed from the entire population we performed a non-parametric Kolmogorov–Smirnov two-sample test between the selected cells (above-threshold) and the whole cell population and visualized the differential abundance using the kernel density plots for each marker. (**Figures 6**, **7**).

In order to obtain the percentage of the CellCnn-selected cells that are concurrent with cells in user-gated population categories (**Figures 6D-F**), we first obtain the set of event numbers of the CellCnn-selected cells denoted by Sc and subsequently perform intersection with the set of event numbers for a particular gated population which provides the set of overlapping cells denoted by So. Finally, the fraction of mapped cell population is obtained by taking ratio between So and Sc. This ratio is obtained for each patient for the following manually-gated populations: Plasmablasts, DN1, DN2, Activated DN, Bnd1, Bnd2, IgM+ IgD- IgM-only, IgM+ IgD+ Mature Naïve, IgM- IgD- Switched Memory, IgM- IgD+ c-delta class switched, IgM+ IgD- IgM Memory, IgM+ IgD+ Pre-switched.

The R programming language was used to implement statistical analyses (91), with supporting visualizations generated in Prism 9.

## Data availability statement

Mass cytometry data (.fcs) presented in the study are deposited at flowrepository.org, experiment ID FR-FCM-Z6YW. Gene expression data tables and additional mass cytometry data tables (raw cell type frequencies, activation marker, and cytokine parameter values) are deposited at data.mendeley.com, accession number: DOI: 10.17632/5mkhzmswyn.1. Original code generated for the analyses in this paper are available at https://medschool.cuanschutz.edu/immunology-and-microbiology/faculty/hsieh/computational-resources.

## Ethics statement

The studies involving humans were approved by Colorado Multiple Institutional Review Board. The studies were conducted in accordance with the local legislation and institutional requirements. Written informed consent for participation in this study was provided by the participants’ legal guardians/next of kin.

## Author contributions

CSW, JCo, and EWYH enrolled the patients on study. RMB, MJS, and EWYH conceived the study, processed all patient samples, developed the reagents, performed experiments, analyzed data, and wrote the manuscript. CSW and JCo performed all the clinical data extraction and analysis. DSK and BMC also performed sample processing and mass cytometry experiments. JEGP assisted in data analysis. RPS and CJ performed batch normalization for mass cytometry experiments. RMB, TG, PR, DP, MC, and DG performed the mass cytometry and gene expression data analysis. MJS, JCa, RR and EWYH provided intellectual guidance for the entire study and manuscript. All authors contributed to the article and approved the submitted version.

## References

[1] Mina R Brunner HI. Pediatric lupus–are there differences in presentation, genetics, response to therapy, and damage accrual compared with adult lupus? Rheum Dis Clin North Am (2010) 36:53–80, vii-viii. doi: 10.1016/j.rdc.2009.12.012 20202591 PMC2837537

[2] Tucker LB Uribe AG Fernandez M Vila LM McGwin G Apte M. Adolescent onset of lupus results in more aggressive disease and worse outcomes: results of a nested matched case-control study within LUMINA, a multiethnic US cohort (LUMINA LVII). Lupus (2008) 17:314–22. doi: 10.1177/0961203307087875 PMC2818044 18413413

[3] Hiraki LT Benseler SM Tyrrell PN Hebert D Harvey E Silverman ED. Clinical and laboratory characteristics and long-term outcome of pediatric systemic lupus erythematosus: a longitudinal study. J Pediatr (2008) 152:550–6. doi: 10.1016/j.jpeds.2007.09.019 18346514

[4] Tunnicliffe DJ Palmer SC Henderson L Masson P Craig JC Tong A. Immunosuppressive treatment for proliferative lupus nephritis. Cochrane Database Syst Rev (2018) 6:CD002922. doi: 10.1002/14651858.CD002922.pub4 29957821 PMC6513226

[5] Demir S Gulhan B Ozen S Celegen K Batu ED Tas N. Long-term renal survival of paediatric patients with lupus nephritis. Nephrol Dial Transplant (2022) 37:1069–77. doi: 10.1093/ndt/gfab152 33826705

[6] Appel GB Contreras G Dooley MA Ginzler EM Isenberg D Jayne D. Mycophenolate mofetil versus cyclophosphamide for induction treatment of lupus nephritis. J Am Soc Nephrol (2009) 20:1103–12. doi: 10.1681/ASN.2008101028 PMC2678035 19369404

[7] Yo JH Barbour TD Nicholls K. Management of refractory lupus nephritis: challenges and solutions. Open Access Rheumatol (2019) 11:179–88. doi: 10.2147/OARRR.S166303 PMC6636187 31372070

[8] Merrill JT Burgos-Vargas R Westhovens R Chalmers A D'Cruz D Wallace DJ. The efficacy and safety of abatacept in patients with non-life-threatening manifestations of systemic lupus erythematosus: results of a twelve-month, multicenter, exploratory, phase IIb, randomized, double-blind, placebo-controlled trial. Arthritis Rheum (2010) 62:3077–87. doi: 10.1002/art.27601 20533545

[9] Furie R Nicholls K Cheng TT Houssiau F Burgos-Vargas R Chen SL. Efficacy and safety of abatacept in lupus nephritis: a twelve-month, randomized, double-blind study. Arthritis Rheumatol (2014) 66:379–89. doi: 10.1002/art.38260 24504810

[10] Rovin BH Furie R Latinis K Looney RJ Fervenza FC Sanchez-Guerrero J. Efficacy and safety of rituximab in patients with active proliferative lupus nephritis: the lupus nephritis assessment with rituximab study. Arthritis Rheum (2012) 64:1215–26. doi: 10.1002/art.34359 22231479

[11] Merrill JT Neuwelt CM Wallace DJ Shanahan JC Latinis KM Oates JC. Efficacy and safety of rituximab in moderately-to-severely active systemic lupus erythematosus: the randomized, double-blind, phase II/III systemic lupus erythematosus evaluation of rituximab trial. Arthritis Rheum (2010) 62:222–33. doi: 10.1002/art.27233 PMC4548300 20039413

[12] Mysler EF Spindler AJ Guzman R Bijl M Jayne D Furie RA. Efficacy and safety of ocrelizumab in active proliferative lupus nephritis: results from a randomized, double-blind, phase III study. Arthritis Rheum (2013) 65:2368–79. doi: 10.1002/art.38037 23740801

[13] Rovin BH van Vollenhoven RF Aranow C Wagner C Gordon R Zhuang Y. Randomized, double-blind, placebo-controlled study to evaluate the efficacy and safety of treatment with sirukumab (CNTO 136) in patients with active lupus nephritis. Arthritis Rheumatol (2016) 68:2174–83. doi: 10.1002/art.39722 PMC5129491 27110697

[14] Bertsias GK Tektonidou M Amoura Z Aringer M Bajema I Berden JH. Joint european league against rheumatism and european renal association-european dialysis and transplant association (EULAR/ERA-EDTA) recommendations for the management of adult and paediatric lupus nephritis. Ann Rheum Dis (2012) 71:1771–82. doi: 10.1136/annrheumdis-2012-201940 PMC3465859 22851469

[15] Floege J Barbour SJ Cattran DC Hogan JJ Nachman PH Tang SCW. Management and treatment of glomerular diseases (part 1): conclusions from a kidney disease: Improving global outcomes (KDIGO) controversies conference. Kidney Int (2019) 95:268–80. doi: 10.1016/j.kint.2018.10.018 30665568

[16] Hahn BH McMahon MA Wilkinson A Wallace WD Daikh DI Fitzgerald JD. American college of rheumatology guidelines for screening, treatment, and management of lupus nephritis. Arthritis Care Res (Hoboken) (2012) 64:797–808. doi: 10.1002/acr.21664 22556106 PMC3437757

[17] Arbuckle MR McClain MT Rubertone MV Scofield RH Dennis GJ James JA. Development of autoantibodies before the clinical onset of systemic lupus erythematosus. N Engl J Med (2003) 349:1526–33. doi: 10.1056/NEJMoa021933 14561795

[18] William J Euler C Christensen S Shlomchik MJ. Evolution of autoantibody responses *via* somatic hypermutation outside of germinal centers. Science (2002) 297:2066–70. doi: 10.1126/science.1073924 12242446

[19] Duty JA Szodoray P Zheng NY Koelsch KA Zhang Q Swiatkowski M. Functional anergy in a subpopulation of naive b cells from healthy humans that express autoreactive immunoglobulin receptors. J Exp Med (2009) 206:139–51. doi: 10.1084/jem.20080611 PMC2626668 19103878

[20] Smith MJ Rihanek M Coleman BM Gottlieb PA Sarapura VD Cambier JC. Activation of thyroid antigen-reactive b cells in recent onset autoimmune thyroid disease patients. J Autoimmun (2018) 89:82–9. doi: 10.1016/j.jaut.2017.12.001 PMC5902436 29233566

[21] Smith MJ Packard TA O'Neill SK Henry Dunand CJ Huang M Fitzgerald-Miller L. Loss of anergic b cells in prediabetic and new-onset type 1 diabetic patients. Diabetes (2015) 64:1703–12. doi: 10.2337/db13-1798 PMC4407867 25524915

[22] Stensland Z. C. Magera C. A. Broncucia H. Gomez B. D. Rios-Guzman N. M. Wells K. L.. Identification of an anergic BND cell-derived activated B cell population (BND2) in young-onset type 1 diabetes patients. J Exp Med (2023) 220(8). doi: 10.1084/jem.20221604 PMC10192302 37184563

[23] Odendahl M Jacobi A Hansen A Feist E Hiepe F Burmester GR. Disturbed peripheral b lymphocyte homeostasis in systemic lupus erythematosus. J Immunol (2000) 165:5970–9. doi: 10.4049/jimmunol.165.10.5970 11067960

[24] Jacobi AM Reiter K Mackay M Aranow C Hiepe F Radbruch A. Activated memory b cell subsets correlate with disease activity in systemic lupus erythematosus: Delineation by expression of CD27, IgD, and CD95. Arthritis Rheumatism (2008) 58:1762–73. doi: 10.1002/art.23498 18512812

[25] Lugar PL Love C Grammer AC Dave SS Lipsky PE. Molecular characterization of circulating plasma cells in patients with active systemic lupus erythematosus. PloS One (2012) 7:e44362. doi: 10.1371/journal.pone.0044362 23028528 PMC3448624

[26] Banchereau R Hong S Cantarel B Baldwin N Baisch J Edens M. Personalized immunomonitoring uncovers molecular networks that stratify lupus patients. Cell (2016) 165:551–65. doi: 10.1016/j.cell.2016.03.008 PMC5426482 27040498

[27] Hiroyuki Y Hideki U. Shared and distinct roles of t peripheral helper and t follicular helper cells in human diseases. Cell Mol Immunol (2021) 18:523–27. doi: 10.1038/s41423-020-00529-z PMC8027819 32868910

[28] Bocharnikov A V. K Vanessa SW Ye C Chamith YF Guoxing W. PD-1hiCXCR5- t peripheral helper cells promote b cell responses in lupus *via* MAF and IL-21. JCI Insight (2019) 4(20):e130062. doi: 10.1172/JCI.INSIGHT.130062 31536480 PMC6824311

[29] Wei C Anolik J Cappione A Zheng B Pugh-Bernard A Brooks J. A new population of cells lacking expression of CD27 represents a notable component of the b cell memory compartment in systemic lupus erythematosus. J Immunol (2007) 178:6624–33. doi: 10.4049/jimmunol.178.10.6624 17475894

[30] Jenks SA Cashman KS Zumaquero E Marigorta UM Patel AV Wang X. Distinct effector b cells induced by unregulated toll-like receptor 7 contribute to pathogenic responses in systemic lupus erythematosus. Immunity (2018) 49:725–39 e6. doi: 10.1016/j.immuni.2018.08.015 30314758 PMC6217820

[31] Szelinski F Stefanski AL Schrezenmeier E Rincon-Arevalo H Wiedemann A Reiter K. Antigen-experienced CXCR5(-) CD19(low) b cells are plasmablast precursors expanded in SLE. Arthritis Rheumatol (2022) 74:1556–68. doi: 10.1002/art.42157 35507291

[32] Djamel N-B Seunghee H Radu M Guo C Mohan B Jeanine B. Mapping systemic lupus erythematosus heterogeneity at the single-cell level. Nat Immunol (2020) 21:1094–106. doi: 10.1038/s41590-020-0743-0 PMC7442743 32747814

[33] Myles A Sanz I Cancro MP. T-bet(+) b cells: A common denominator in protective and autoreactive antibody responses? Curr Opin Immunol (2019) 57:40–5. doi: 10.1016/j.coi.2019.01.002 PMC8356139 30784957

[34] Slutsky RA Higgins CB. Thallium scintigraphy in experimental toxic pulmonary edema: relationship to extravascular pulmonary fluid. J Nucl Med (1984) 25:581–91. 6726437

[35] Rubtsova K Rubtsov AV Cancro MP Marrack P. Age-associated b cells: A t-bet-Dependent effector with roles in protective and pathogenic immunity. J Immunol (2015) 195:1933–7. doi: 10.4049/jimmunol.1501209 PMC4548292 26297793

[36] Karnell JL Kumar V Wang J Wang S Voynova E Ettinger R. Role of CD11c(+) t-bet(+) b cells in human health and disease. Cell Immunol (2017) 321:40–5. doi: 10.1016/j.cellimm.2017.05.008 28756897

[37] Arazi A Rao DA Berthier CC Davidson A Liu Y Hoover PJ. The immune cell landscape in kidneys of patients with lupus nephritis. Nat Immunol (2019) 20:902–14. doi: 10.1038/s41590-019-0398-x PMC6726437 31209404

[38] Wenderfer SE Ruth NM Brunner HI. Advances in the care of children with lupus nephritis. Pediatr Res (2017) 81:406–14. doi: 10.1038/pr.2016.247 27855151

[39] Der E Ranabothu S Suryawanshi H Akat KM Clancy R Morozov P. Single cell RNA sequencing to dissect the molecular heterogeneity in lupus nephritis. JCI Insight (2017) 2(9):e93009. doi: 10.1172/jci.insight.93009 28469080 PMC5414553

[40] Der E Suryawanshi H Morozov P Kustagi M Goilav B Ranabothu S. Tubular cell and keratinocyte single-cell transcriptomics applied to lupus nephritis reveal type i IFN and fibrosis relevant pathways. Nat Immunol (2019) 20:915–27. doi: 10.1038/s41590-019-0386-1 PMC6584054 31110316

[41] Fava A Rao DA Mohan C Zhang T Rosenberg A Fenaroli P. Urine proteomics and renal single-cell transcriptomics implicate interleukin-16 in lupus nephritis. Arthritis Rheumatol (2022) 74:829–39. doi: 10.1002/art.42023 PMC9050800 34783463

[42] Ardoin SP Daly RP Merzoug L Tse K Ardalan K Arkin L. Research priorities in childhood-onset lupus: results of a multidisciplinary prioritization exercise. Pediatr Rheumatol Online J (2019) 17:32. doi: 10.1186/s12969-019-0327-4 31262324 PMC6600895

[43] Mina R von Scheven E Ardoin SP Eberhard BA Punaro M Ilowite N. Consensus treatment plans for induction therapy of newly diagnosed proliferative lupus nephritis in juvenile systemic lupus erythematosus. Arthritis Care Res (Hoboken) (2012) 64:375–83. doi: 10.1002/acr.21558 PMC3457803 22162255

[44] Guthridge JM Lu R Tran LT Arriens C Aberle T Kamp S. Adults with systemic lupus exhibit distinct molecular phenotypes in a cross-sectional study. EClinicalMedicine (2020) 20:100291. doi: 10.1016/j.eclinm.2020.100291 32154507 PMC7058913

[45] O'Gorman WE Kong DS Balboni IM Rudra P Bolen CR Ghosh D. Mass cytometry identifies a distinct monocyte cytokine signature shared by clinically heterogeneous pediatric SLE patients. J Autoimmun (2017) S0896-8411(16):30412–7. doi: 10.1016/j.jaut.2017.03.010 PMC5628110 28389038

[46] Rudra P Baxter R Hsieh EWY Ghosh D. Compositional data analysis using kernels in mass cytometry data. Bioinform Adv (2022) 2:vbac003. doi: 10.1093/bioadv/vbac003 35224501 PMC8867823

[47] Wangriatisak K Thanadetsuntorn C Krittayapoositpot T Leepiyasakulchai C Suangtamai T Ngamjanyaporn P. The expansion of activated naive DNA autoreactive b cells and its association with disease activity in systemic lupus erythematosus patients. Arthritis Res Ther (2021) 23:179. doi: 10.1186/s13075-021-02557-0 34229724 PMC8259008

[48] Tipton CM Fucile CF Darce J Chida A Ichikawa T Gregoretti I. Diversity, cellular origin and autoreactivity of antibody-secreting cell population expansions in acute systemic lupus erythematosus. Nat Immunol (2015) 16:755–65. doi: 10.1038/ni.3175 PMC4512288 26006014

[49] Smith MJ Rihanek M Wasserfall C Mathews CE Atkinson MA Gottlieb PA. Loss of b-cell anergy in type 1 diabetes is associated with high-risk HLA and non-HLA disease susceptibility alleles. Diabetes (2018) 67:697–703. doi: 10.2337/db17-0937 29343548 PMC5860860

[50] Smith MJ Ford BR Rihanek M Coleman BM Getahun A Sarapura VD. Elevated PTEN expression maintains anergy in human b cells and reveals unexpectedly high repertoire autoreactivity. JCI Insight (2019) 4(3):e123384. doi: 10.1172/jci.insight.123384 30728334 PMC6413793

[51] Stensland ZC Coleman BM Rihanek M Baxter RM Gottlieb PA Hsieh EWY. Peripheral immunophenotyping of AITD subjects reveals alterations in immune cells in pediatric vs adult-onset AITD. iScience (2022) 25:103626. doi: 10.1016/j.isci.2021.103626 35005561 PMC8718984

[52] Stensland ZC Magera CA Broncucia H Gomez BD Rios-Guzman NM. Identification of an anergic BND cell–derived activated B cell population (BND2) in young-onset type 1 diabetes patients. J Exp Med (2023) 220(8):e20221604. doi: 10.1084/jem.20221604 37184563 PMC10192302

[53] Arvaniti E Claassen M. Sensitive detection of rare disease-associated cell subsets *via* representation learning. Nat Commun (2017) 8:14825. doi: 10.1038/ncomms14825 28382969 PMC5384229

[54] Galli E Hartmann FJ Schreiner B Ingelfinger F Arvaniti E Diebold M. GM-CSF and CXCR4 define a t helper cell signature in multiple sclerosis. Nat Med (2019) 25:1290–300. doi: 10.1038/s41591-019-0521-4 PMC6689469 31332391

[55] Gagro A Servis D Cepika AM Toellner KM Grafton G Taylor DR. Type i cytokine profiles of human naive and memory b lymphocytes: a potential for memory cells to impact polarization. Immunology (2006) 118:66–77. doi: 10.1111/j.1365-2567.2006.02342.x 16630024 PMC1782263

[56] Duddy ME Alter A Bar-Or A. Distinct profiles of human b cell effector cytokines: a role in immune regulation? J Immunol (2004) 172:3422–7. doi: 10.4049/jimmunol.172.6.3422 15004141

[57] Nashi E Wang Y Diamond B. The role of b cells in lupus pathogenesis. Int J Biochem Cell Biol (2010) 42:543–50. doi: 10.1016/j.biocel.2009.10.011 PMC2835836 19850148

[58] Andreoli L Bertsias GK Agmon-Levin N Brown S Cervera R Costedoat-Chalumeau N. EULAR recommendations for women's health and the management of family planning, assisted reproduction, pregnancy and menopause in patients with systemic lupus erythematosus and/or antiphospholipid syndrome. Ann Rheum Dis (2017) 76:476–85. doi: 10.1136/annrheumdis-2016-209770 PMC5446003 27457513

[59] Oon S Huq M Golder V Ong PX Morand EF Nikpour M. Lupus low disease activity state (LLDAS) discriminates responders in the BLISS-52 and BLISS-76 phase III trials of belimumab in systemic lupus erythematosus. Ann Rheum Dis (2019) 78:629–33. doi: 10.1136/annrheumdis-2018-214427 30679152

[60] Morand EF Trasieva T Berglind A Illei GG Tummala R. Lupus low disease activity state (LLDAS) attainment discriminates responders in a systemic lupus erythematosus trial: post-hoc analysis of the phase IIb MUSE trial of anifrolumab. Ann Rheum Dis (2018) 77:706–13. doi: 10.1136/annrheumdis-2017-212504 PMC5909750 29420200

[61] Jones-Leone A Flint S Levy R Roth D Henderson R Wilkinson C. Efficacy analysis of patients with systemic lupus erythematosus treated with belimumab or placebo plus standard therapy in phase 3 trials by baseline levels of BLyS mRNA and type 1 interferon inducible gene signature status. Arthritis Rheumatol (2019) 71.

[62] Ronnblom L Leonard D. Interferon pathway in SLE: one key to unlocking the mystery of the disease. Lupus Sci Med (2019) 6:e000270. doi: 10.1136/lupus-2018-000270 31497305 PMC6703304

[63] Andreakos E Zanoni I Galani IE. Lambda interferons come to light: dual function cytokines mediating antiviral immunity and damage control. Curr Opin Immunol (2019) 56:67–75. doi: 10.1016/j.coi.2018.10.007 30399529 PMC6541392

[64] Ronnblom L Eloranta ML. The interferon signature in autoimmune diseases. Curr Opin Rheumatol (2013) 25:248–53. doi: 10.1097/BOR.0b013e32835c7e32 23249830

[65] Lazear HM Schoggins JW Diamond MS. Shared and distinct functions of type i and type III interferons. Immunity (2019) 50:907–23. doi: 10.1016/j.immuni.2019.03.025 PMC6839410 30995506

[66] Pollard KM Cauvi DM Toomey CB Morris KV Kono DH. Interferon-gamma and systemic autoimmunity. Discovery Med (2013) 16:123–31. PMC3934799 23998448

[67] Lioulios G Mitsoglou Z Fylaktou A Xochelli A Christodoulou M Stai S. Exhausted but not senescent t lymphocytes predominate in lupus nephritis patients. Int J Mol Sci (2022) 23:13928. doi: 10.3390/ijms232213928 36430418 PMC9694088

[68] Yuan S Zeng Y Li J Wang C Li W He Z. Phenotypical changes and clinical significance of CD4(+)/CD8(+) t cells in SLE. Lupus Sci Med (2022) 9:e000660. doi: 10.1136/lupus-2022-000660 35732344 PMC9226979

[69] Hurtado C Rojas-Gualdron DF Urrego R Cashman K Vasquez-Trespalacios EM Diaz-Coronado JC. Altered b cell phenotype and CD27+ memory b cells are associated with clinical features and environmental exposure in colombian systemic lupus erythematosus patients. Front Med (Lausanne) (2022) 9:950452. doi: 10.3389/fmed.2022.950452 36148466 PMC9485945

[70] Cassia M Alberici F Gallieni M Jayne D. Lupus nephritis and b-cell targeting therapy. Expert Rev Clin Immunol (2017) 13:951–62. doi: 10.1080/1744666X.2017.1366855 28800401

[71] Sanz I Wei C Jenks SA Cashman KS Tipton C Woodruff MC. Challenges and opportunities for consistent classification of human b cell and plasma cell populations. Front Immunol (2019) 10:2458. doi: 10.3389/fimmu.2019.02458 31681331 PMC6813733

[72] Wang S Wang J Kumar V Karnell JL Naiman B Gross PS. IL-21 drives expansion and plasma cell differentiation of autoreactive CD11c(hi)T-bet(+) b cells in SLE. Nat Commun (2018) 9:1758. doi: 10.1038/s41467-018-03750-7 29717110 PMC5931508

[73] Ping Min C George CT. T cell abnormalities in the pathogenesis of systemic lupus erythematosus: an update. Curr Rheumatol Rep (2021) 23:12. doi: 10.1007/S11926-020-00978-5 33512577 PMC8601587

[74] Soni C Perez OA Voss WN Pucella JN Serpas L Mehl J. Plasmacytoid dendritic cells and type i interferon promote extrafollicular b cell responses to extracellular self-DNA. Immunity (2020) 52:1022–38 e7. doi: 10.1016/j.immuni.2020.04.015 32454024 PMC7306002

[75] Pena Nunez D. The role of interferon α in human t peripheral helper cells and t follicular helper cells. MS, Harvard: Master's thesis, Harvard Medical School (2020).

[76] Loncharich MF Anderson CW. Interferon inhibition for lupus with anifrolumab: Critical appraisal of the evidence leading to FDA approval. ACR Open Rheumatol (2022) 4:486–91. doi: 10.1002/acr2.11414 PMC9190216 35157371

[77] Morand EF Furie R Tanaka Y Bruce IN Askanase AD Richez C. Trial of anifrolumab in active systemic lupus erythematosus. N Engl J Med (2020) 382:211–21. doi: 10.1056/NEJMoa1912196 31851795

[78] Bruce IN Furie RA Morand EF Manzi S Tanaka Y Kalunian KC. Concordance and discordance in SLE clinical trial outcome measures: analysis of three anifrolumab phase 2/3 trials. Ann Rheum Dis (2022) 81:962–69. doi: 10.1136/annrheumdis-2021-221847 PMC9213793 35580976

[79] David J Brad R Eduardo FM Richard AF Frederic AH Teodora T. Phase II randomised trial of type i interferon inhibitor anifrolumab in patients with active lupus nephritis. Ann Rheumatic Dis (2022) 81:496. doi: 10.1136/annrheumdis-2021-221478 PMC8921596 35144924

[80] Hochberg MC. Updating the american college of rheumatology revised criteria for the classification of systemic lupus erythematosus. Arthritis Rheum (1997) 40:1725. doi: 10.1002/art.1780400928 9324032

[81] Gladman DD Ibanez D Urowitz MB. Systemic lupus erythematosus disease activity index 2000. J Rheumatol (2002) 29:288–91. 11838846

[82] Franklyn K Lau CS Navarra SV Louthrenoo W Lateef A Hamijoyo L. Definition and initial validation of a lupus low disease activity state (LLDAS). Ann Rheum Dis (2016) 75:1615–21. doi: 10.1136/annrheumdis-2015-207726 26458737

[83] Baxter RM Kong DS Garcia-Perez JE O'Gorman WE Hsieh EWY. Single-cell analysis of immunophenotype and cytokine production in peripheral whole blood *via* mass cytometry. J Vis Exp (2018) 136:57780. doi: 10.3791/57780 PMC6101995 30010641

[84] Kim CH Abedi M Liu Y Panuganti S Flores F Shah KR. A novel technology for multiplex gene expression analysis directly from whole blood samples stabilized at ambient temperature using an RNA-stabilizing buffer. J Mol Diagn (2015) 17:118–27. doi: 10.1016/j.jmoldx.2014.11.002 25684272

[85] Mann HB Whitney DR. On a test of whether one of two random variables is stochastically larger than the other. Ann Math Stat (1947) 18:50–60. doi: 10.1214/aoms/1177730491

[86] Ward JH. Hierarchical grouping to optimize an objective function. J Am Stat Assoc (1963) 58:236–44. doi: 10.1080/01621459.1963.10500845

[87] Benjamini Y Hochberg Y. Controlling the false discovery rate: A practical and powerful approach to multiple testing. J R Stat Society: Ser B (Methodological) (1995) 57:289–300. doi: 10.1111/j.2517-6161.1995.tb02031.x

[88] Nowicka M Krieg C Crowell HL Weber LM Hartmann FJ Guglietta S. CyTOF workflow: differential discovery in high-throughput high-dimensional cytometry datasets. F1000Res (2017) 6:748. doi: 10.12688/f1000research.11622.3 28663787 PMC5473464

[89] Aitchison J Barceló-Vidal C Martín-Fernández JA Pawlowsky-Glahn V. Logratio analysis and compositional distance. Math Geology (2000) 32:271–75. doi: 10.1023/A:1007529726302

[90] Kingma DP Ba J. Adam: A method for stochastic optimization. arXiv (2014). doi: 10.48550/ARXIV.1412.6980

[91] Team RC. R: A language and environment for statistical computing. Vienna, Austria: R Foundation for Statistical Computing (2022). Available at: https://www.R-project.org/.

