# Supplementary material for: Expansion of extrafollicular B and T cell subsets in childhood-onset systemic lupus erythematosus
Source: Front Immunol. 2023 Oct 27;14:1208282. doi: 10.3389/fimmu.2023.1208282 (PMC10641733; doi:10.3389/fimmu.2023.1208282)
Supplement: Supplementary file 1 [file DataSheet_1.pdf]

Supplemental Figure 1

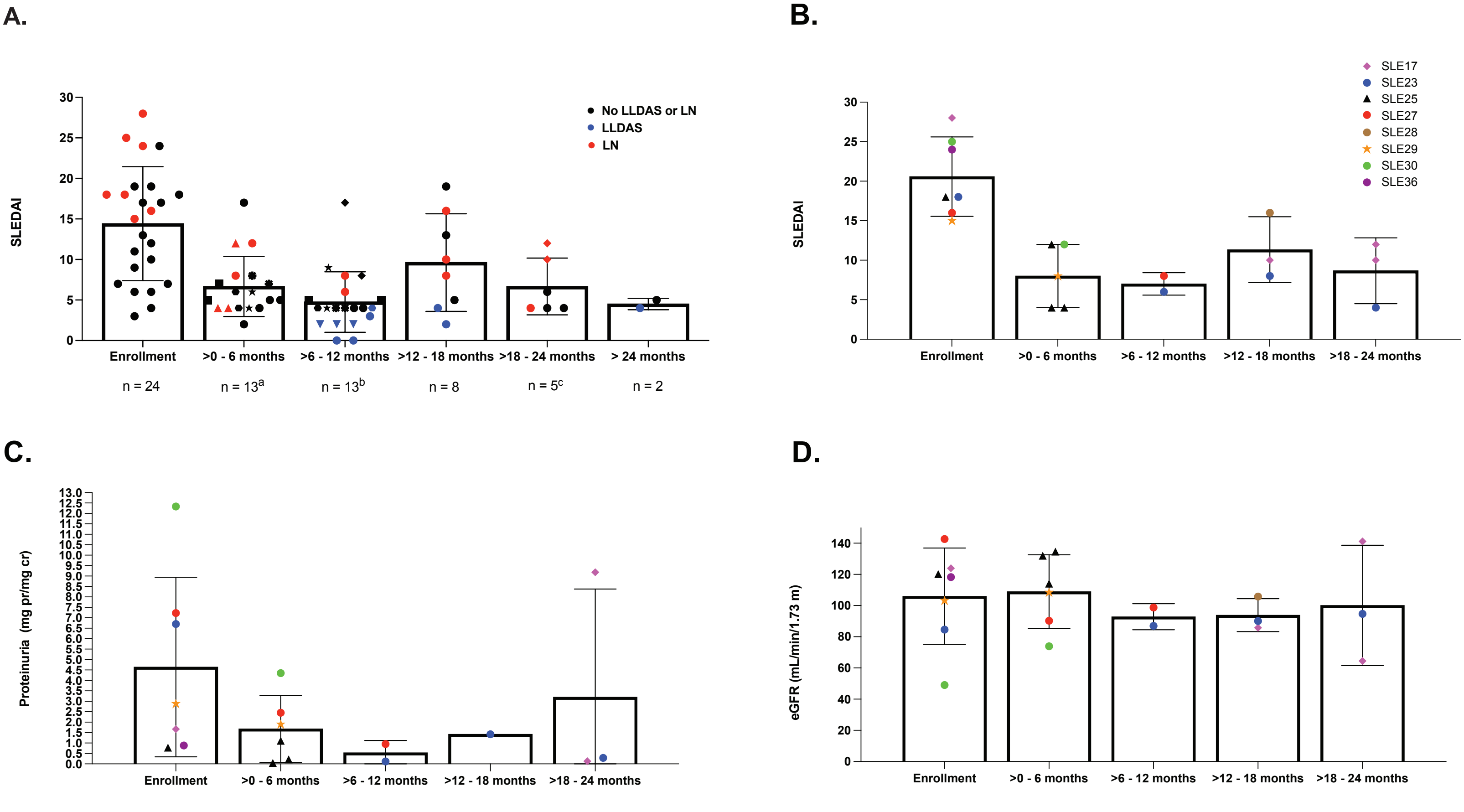

Supplemental Figure 1. cSLE and cLN patient cohort disease activity scores and renal metrics across the three-year longitudinal study period. A. cSLE cohort disease activity scores (SLEDAI). n = number of unique patients with sample(s) during the follow up time period indicated. LLADS = lupus low disease activity state. Red shapes indicate patients with lupus nephritis (LN). Blue shapes indicate patients who reached LLDAS during that follow up time period. Black shapes indicate patients who did not reach LLDAS and did not have LN. Circles designate unique patients within a time period. Patients with multiple samples within the same time period are indicated by a particular shape within that period. [a]Five individuals with multiple samples during the period. [b]Six individuals with multiple samples during the period. [c]One individual with multiple samples during the period. **B.** cLN cohort disease activity scores (SLEDAI) over time. **C.** Urine protein:creatinine ratio (UPCR) change over time. **D.** Estimated glomerular filtration rate (eGFR) change over time. An identical color and shape is used to represent a singular patient over time. Some patients have multiple samples during a given period.

Supplemental Figure 2

A. IFN-1 module single gene expression comparisons

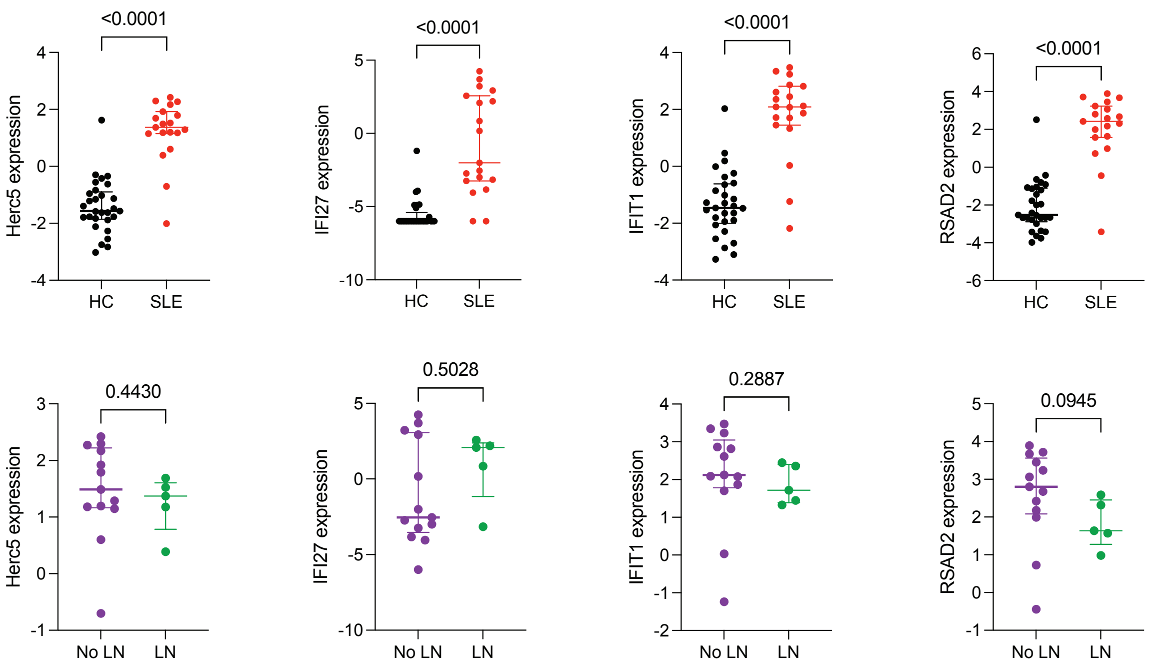

B. IFN-γ module single gene expression comparisons

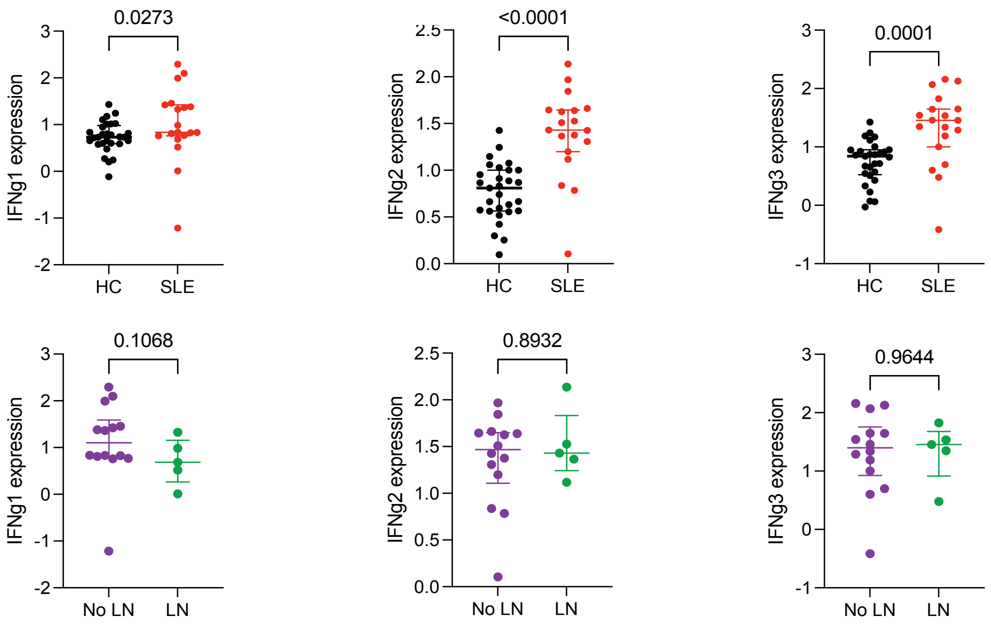

Supplemental Figure 2

C. Plasmablast module single gene expression comparisons

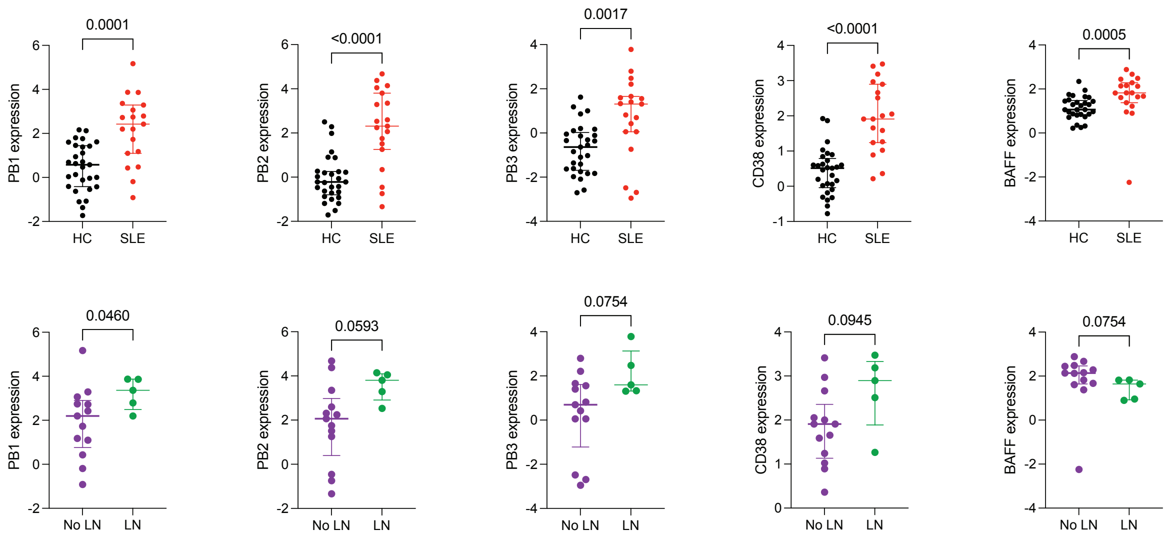

D. T cell activation/exhaustion single gene expression comparisons

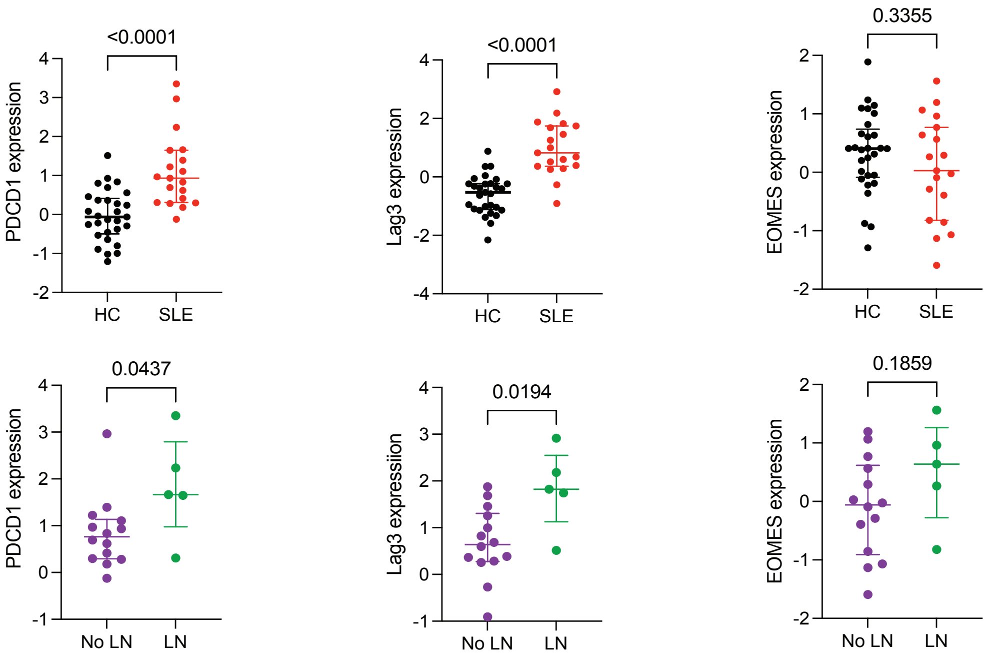

Supplemental Figure 2: Untreated cSLE patients demonstrate enhanced single gene mRNA expression within IFN-1, IFN-, Plasmablast, and T cell exhaustion modules compared HC. Gene expression analysis of HC (black, n=23) and SLE (red, n=22) subjects without LN (purple, n=15) or with LN (green, n=7) at time of diagnosis. **A.** IFN-1 module genes Herc5, IFI27, IFIT1, and RSAD5 shown for HC (black) vs. SLE (red) and SLE with (green)/without LN (purple) at time of diagnosis. **B.** IFN- module genes IFN- 1, 2, and 3 (proprietary) shown for HC v. SLE (top) and SLE with/without LN at time of diagnosis (bottom). **C.** Plasmablast module genes PB 1, 2, and 3 (proprietary), CD38, and BAFF shown for HC v. SLE (top) and SLE with/without LN at time of diagnosis (bottom). **D.** T activation/ex-haustion-related genes PD1, Lag3, and EOMES shown for HC v. SLE (top) and SLE with/without LN at time of diagnosis (bottom). p values shown within each module comparison. Median ± SEM shown. p-values shown within each module comparison determined by Mann-Whitney U test with FDR correction; signficance <0.05.

Supplemental Figure 3

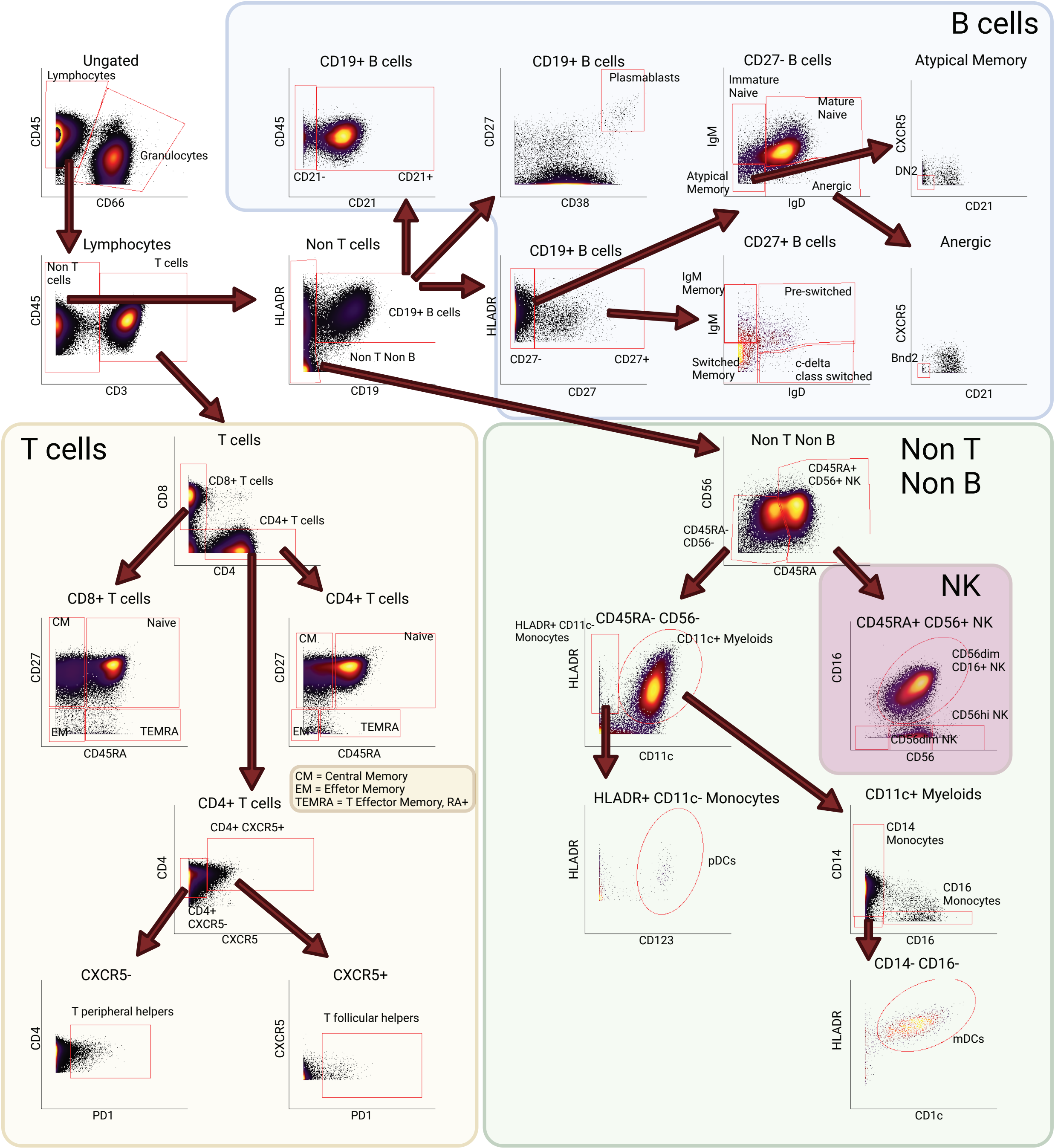

Supplemental Figure 2. Gating strategy to delineate major lymphoid and myeloid cell subsets.
(Created with BioRender.com)

Supplemental Figure 4

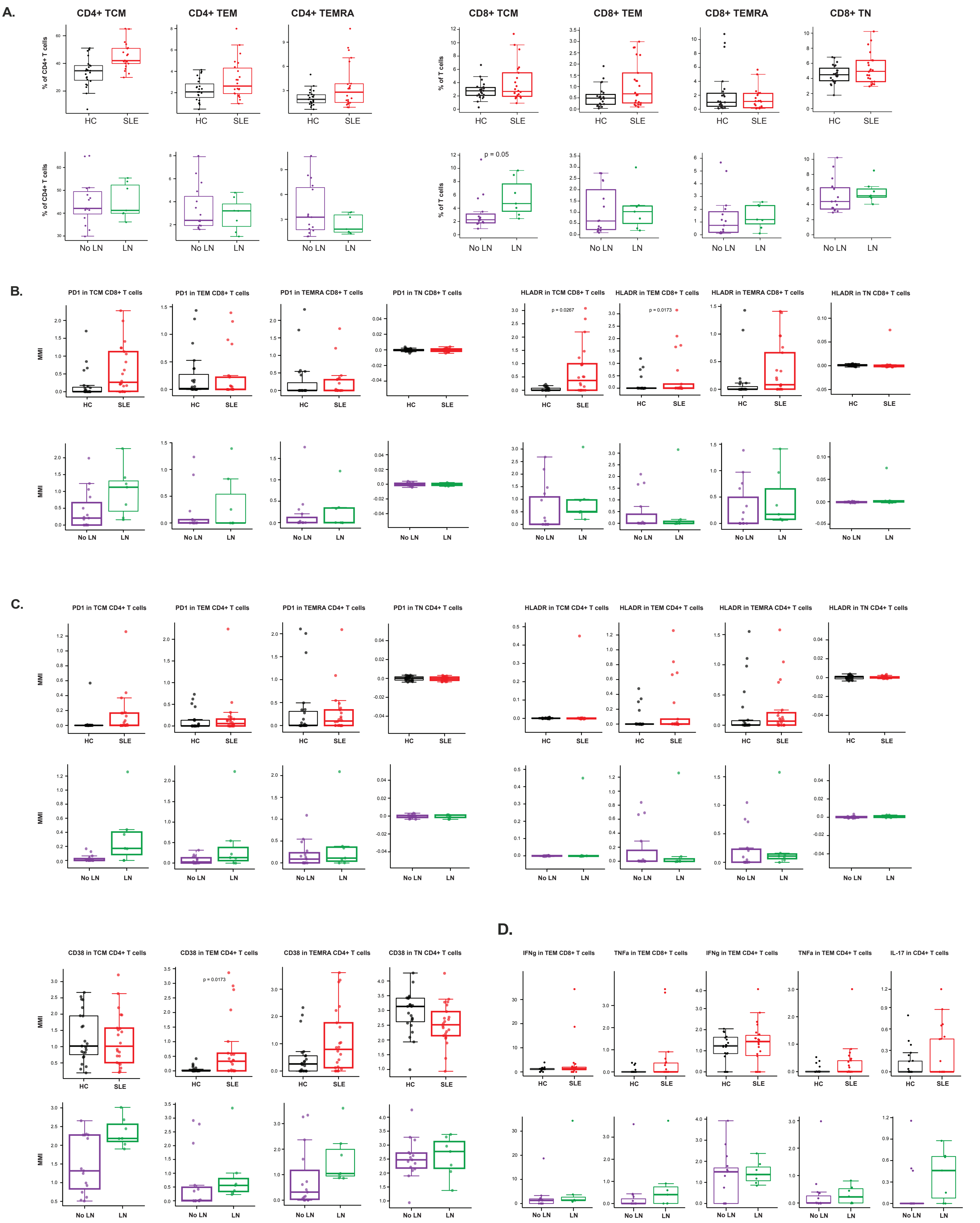

Supplemental Figure 4 ● No LN ● LN

E.

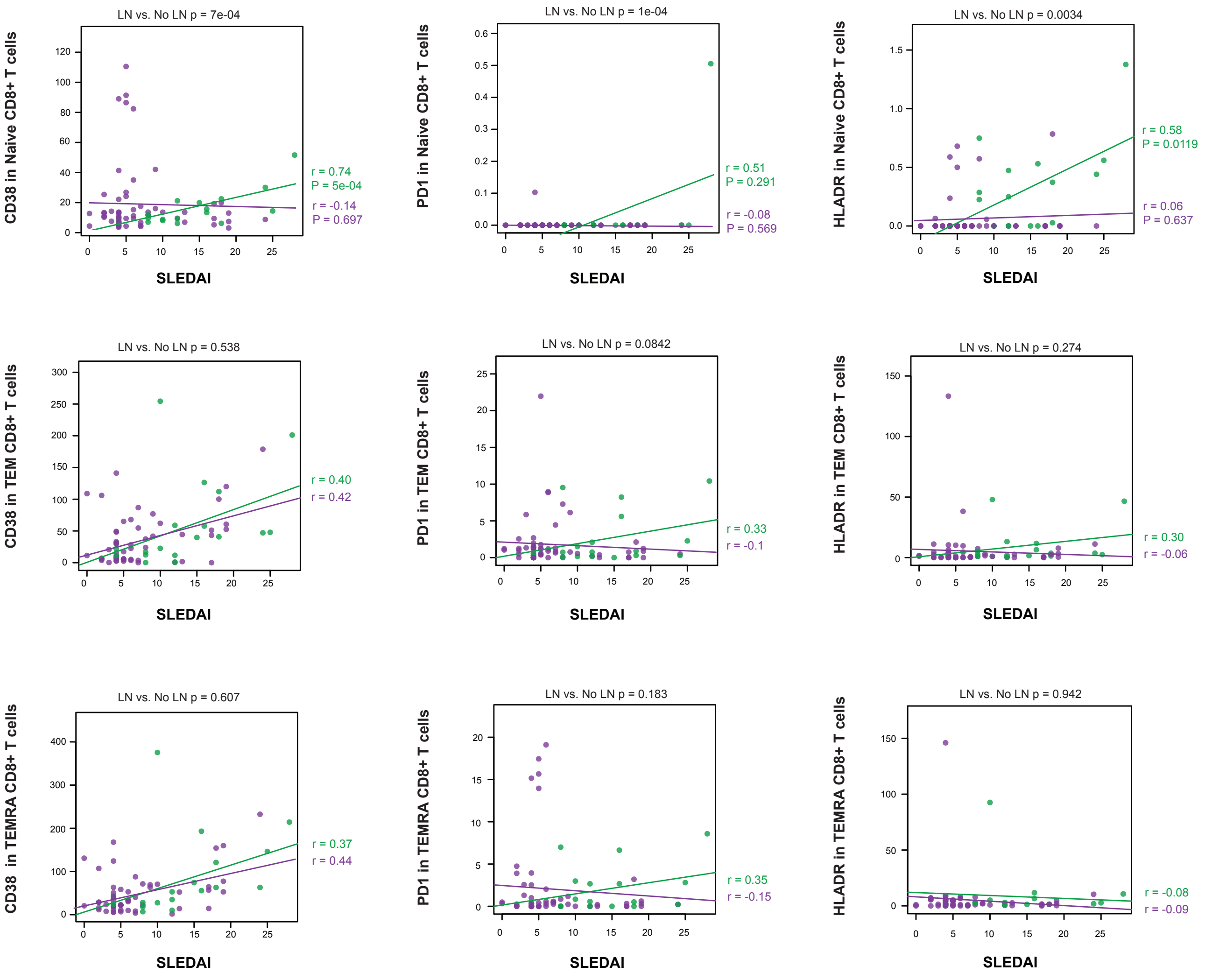

F.

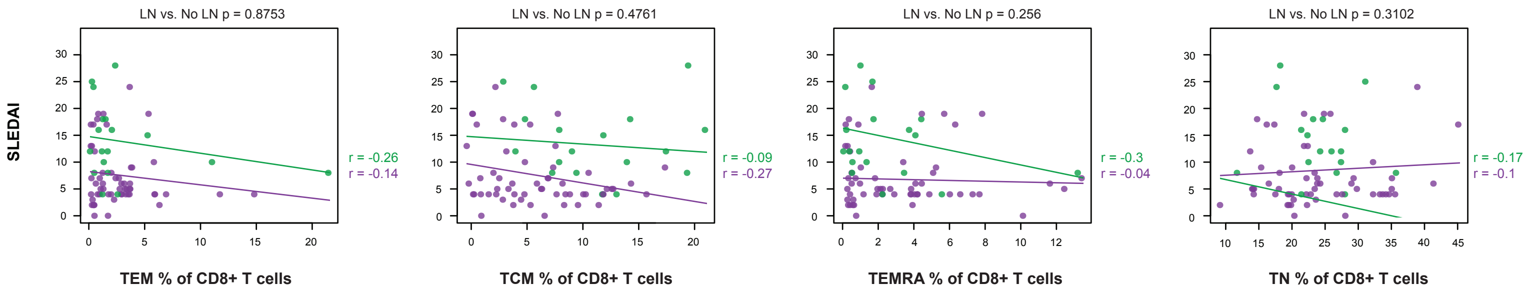

Supplemental Figure 3: CD4+ T cells subset frequencies, T cell activation marker expression, T cell cytokine production, and correlations of surface activation marker expression on CD8+ T cell subsets and CD8+ T cell subset frequency vs. SLEDAI score. Mass cytometry analysis of HC (black, n=23) and SLE (red, n=22) subjects without LN (purple, n=15) or with LN (green, n=7) at time of diagnosis (A-D) or longitudinally (E,F). **A.** Non-naive CD4+ subset (TCM, TEM, TEMRA) frequencies as % of CD4+ T cells for HC (black) vs. SLE (red) and SLE with (green)/without LN (purple) at time of diagnosis and CD8+ T cell subsets as a % of total T cells. NS for all plots, except for TCM in No LN vs. LN (p = 0.05). **B.** Mean metal intensity (MMI) for PD-1 and HLADR on CD8+ subsets (TCM, TEM, TEMRA, TN) for HC (black) vs. SLE (red) and SLE with (green)/without LN (purple). **C.** Mean metal intensity (MMI; arcsin transformed) for PD-1, HLADR, and CD38 on CD4+ subsets (TCM, TEM, TEMRA, TN) or HC (black) vs. SLE (red) and SLE with (green)/without LN (purple). **D.** Mean metal intensity (MMI; arcsin transformed) for IFN- and TNF- on effector subsets (CD8+ and CD4+ TEM) and IL-17A on all CD4+ cells for HC (black) vs. SLE (red) and SLE with (green)/without LN (purple). For all box plots (median, Q1, Q3) p-values shown within each module comparison determined by Kruskal Wallace test with FDR correction; signficance <0.05. **E.** Correlations of surface activation marker expression (CD38, PD1, and HLADR untransformed MMI) on CD8+ TN, TEM, and TEMRA subsets vs. SLEDAI score (x-axis) for SLE subjects at all timepoints, accounting for repeated measures. **F.** Correlations of CD8+ subset (TCM, TEM, TEMRA, TN) frequency out of total CD8+ cells vs. SLEDAI score (y-axis) for SLE subjects at all timepoints, accounting for repeated measures. p values at top of plots test differential correlations between No LN (purple, n=48) and LN (green, n=16) r and p values at right of plots describe No LN and LN specific correlations. Correlation tests performed by linear mixed model (**methods**).

Supplemental Figure 5

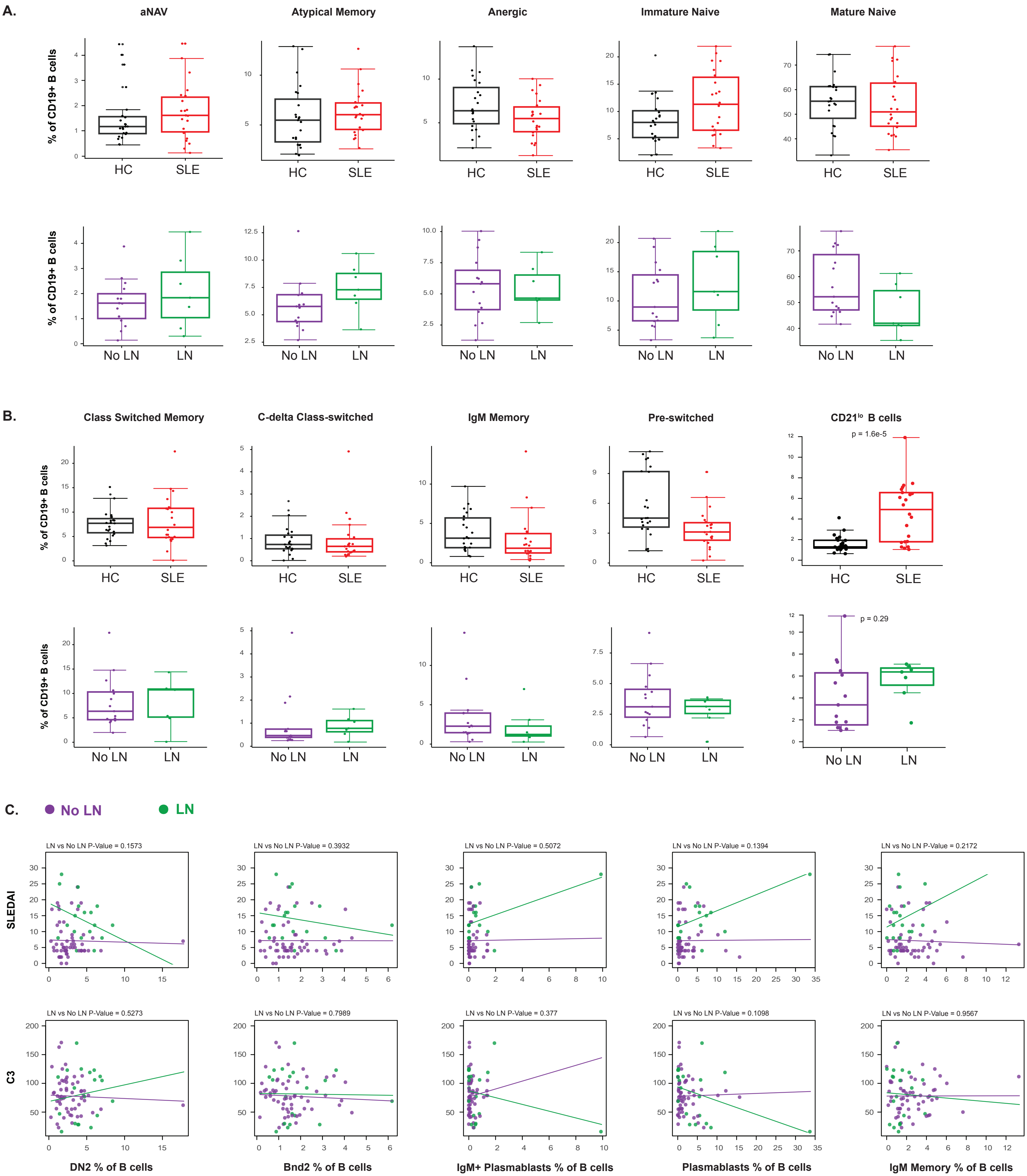

Supplemental Figure 4: $CD27^-$ and CD27+ B cell subset frequencies and correlations of select B cell populations vs. SLEDAI score. Mass cytometry analysis of HC (black, n=23) and SLE (red, n=22) subjects without LN (purple, n=15) or with LN (green, n=7) at time of diagnosis. **A.** $CD27^-$ subsets (aNAV, Atypical Memory, Anergic, IgM-only, Mature Naive) frequencies as % of CD19+ B cells for HC (black) vs. SLE (red) and SLE with (green)/without LN (purple). **B.** $CD27^+$ subsets (Switched Memory, C-delta class-switched, IgM^+ Memory, Pre-switched) frequencies as % of CD19+ B cells for HC (black) vs. SLE (red) and SLE with (green)/ without LN (purple). **C.** CD21lo frequency as % of CD19+ B cells for HC (black) vs. SLE (red) and SLE with (green)/without LN (purple). For all box plots (median, Q1, Q3) p-values shown within each module comparison determined by Kruskal Wallace test with FDR correction; signficance <0.05. **D.** Correlations of population frequency as a % of CD19+ B cells for DN2, Bnd2, IgM+ Plasmablasts, Plasmab-lasts, and IgM^+ Memory vs. SLEDAI score (top) and C3 (mg/dL; bottom) for SLE subjects at all timepoints, accounting for repeated measures. p values at top of plots test differential correlations between No LN (purple, n=48) and LN (green, n=16) r and p values at right of plots describe No LN and LN specific correlations. Correlation tests performed by linear mixed model (**methods**).

Supplemental Figure 6

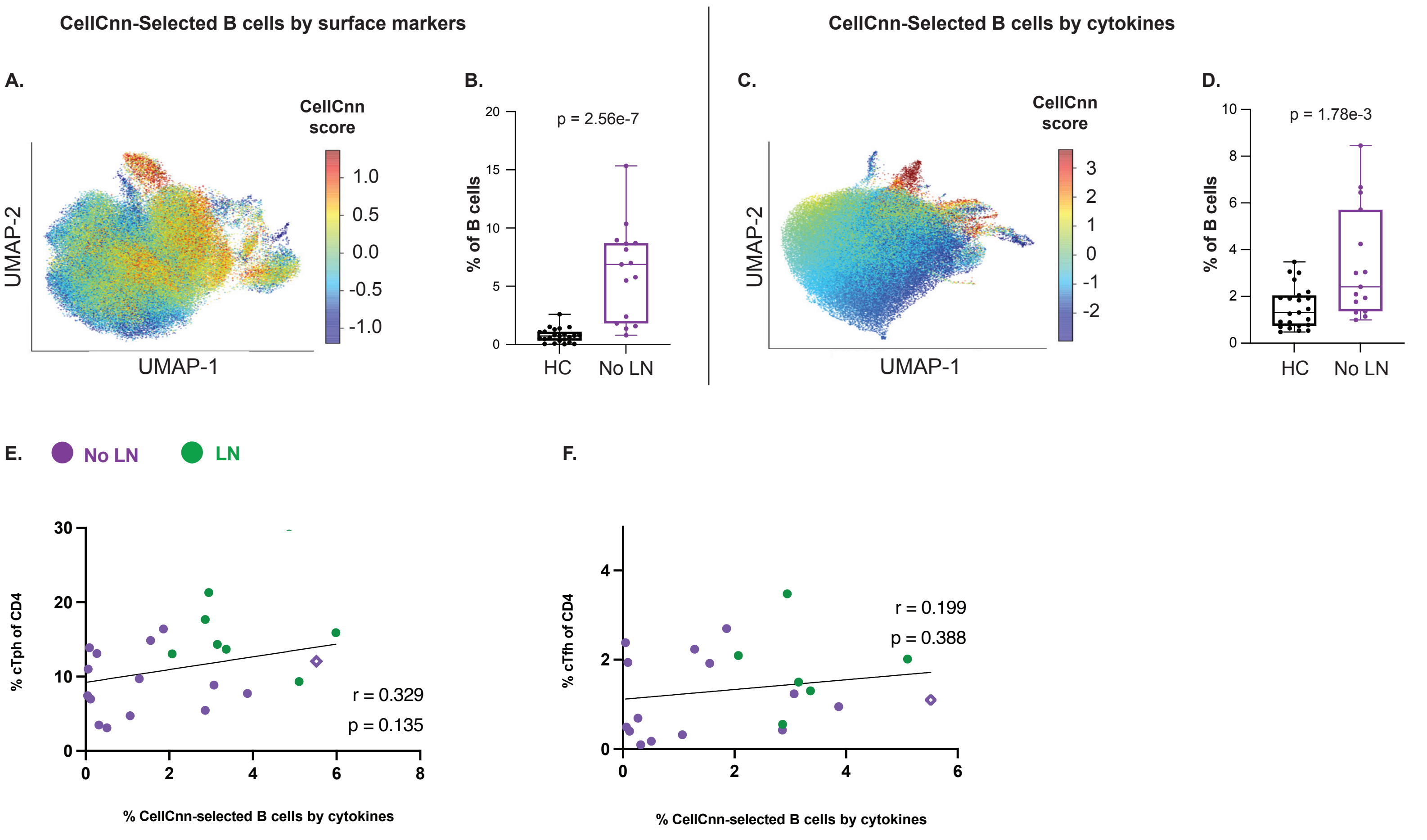

Supplemental Figure 5. The B cell compartment of untreated cSLE patients without LN demonstrates disease-specific signature populations defined by both surface marker expression and cytokine production, but frequency of the cytokine production signature population does not correlate with frequency of cTph or cTfh. Mass cytometry analysis of HC (black, n=23) and cSLE without LN (purple, n=15) subjects at time of diagnosis. A supervised neural network-based learning algorithm (CellCnn) was applied to analyze B cells from HC and SLE at time of diagnosis to identify i) an immunophenotypic signature based on surface markers (**A, B**) and ii) an intracellular cytokine signature based on cytokine expression only (**C, D**) that discriminates between HC and SLE without LN. **A, C.** UMAP projection composite for all samples showing 98th percentile of cells with CellCnn scores conforming to filter (see methods); CellCnn Score coloration scale indicates strength of conformity to cell-selection signature. **B, D.** Frequency of selected cells in HC and SLE subjects without LN. **E, F.** Spearman correlations (significance <0.05) of cTph (**E**) and cTfh (**F**) frequencies vs. CellCnn-selected B cells based on cytokine production for all untreated SLE subjects at time of study enrollment; LN (purple, n=15) and LN (green, n=7). r and p values describe correlation for total cSLE cohort.

Supplemental Table 1: Baseline Clinical Characteristics (n = 24)	
SLEDAI-2K at enrollment[a], median (IQR)	14.5 (8.0 - 18.5)
Cutaneous manifestations[b], n (%)	13 (54.2)
Cytopenia, n (%)	13 (54.2)
Leukopenia, n (%)	9 (37.5)
Thrombocytopenia, n (%)	7 (29.2)
Vasculitis, n (%)	3 (12.5)
Arthritis, n (%)	16 (66.7)
Myositis, n (%)	4 (16.7)
Serositis, n (%)	7 (29.2)
Seizure, n (%)	1 (4.5)
Low complement, n (%)	20 (83.3)
Elevated dsDNA, n (%)	21 (87.5)
Fever, n (%)	6 (25.0)
Nephrotic range proteinuria, n (%)	4/7 (57.1)
Hematuria present, n (%)	6/7 (85.7)
Pyuria present, n (%)	2/7 (28.6)
Urinary cast present, n (%)	1/7 (14.3)

[a]Psychosis, Organic brain syndrome, Vision disturbance, Cranial nerve disorder, Lupus headache, and/or Cerebrovascular accident did not occur

in the cohort

[b]Rash, Alopecia, and/or Oral Ulcers

Supplemental Table 2. Medication(s) use by study participants (n = 20[a])	
Glucocorticoids, n (%)	
Prednisone, oral	18 (90%)
Methylprednisolone, IV	15 (75%)
Antimalarials	20 (100%)
Azathioprine	4 (20%)
Mycophenolate Mofetil/Mycophenolic Acid	13 (65%)
Cyclophosphamide	3 (15%)
Rituximab	4 (20%)
Belimumab	1 (5%)
Methotrexate	4 (20%)
Tacrolimus	1 (5%)
Abatacept	1 (5%)

[a]Four patients in the cohort had a baseline assessment only and were not on medications

Supplemental Table 3. Mass cytometry antibody staining panel.
CellCnn surface: marker was used in surface marker expression analysis (Figure 6)
CellCnn cytokine: marker was used in cytokine production analysis (Figure 7)

Antibody	Clone	Metal	Channel	Titer (Vol/rxn)	Manufacturer	CellCnn surface	CellCnn cytokine
CD45	HI30	Y	89	0.3	Fluidigm 3089003B	X	
CD66	B1.1/CD66	In	113	0.3	BD 551354	X	
CD15	HI98	In	115	0.3	BD 555400	X	
CD21	Bu32	Pr	141	1	Biolegend 354902	X	
CD8	SK1	Nd	142	0.5	Biolegend 344702	X	
CD123	9F5	Nd	143	1	BD 555642	X	
CD3	UCHT1	Nd	144	1	BD 555329	X	
IFN-γ	4S.B3	Nd	145	0.75	Biolegend 502502		X
IgD	IA6-2	Nd	146	1.2	Biolegend 348235	X	
IL-1 α	364-3B3-14	Sm	147	2	Biolegend 500104		X
IL-17a	BL168	Nd	148	2	Biolegend 512302		X
CD7	M-T701	Sm	149	1	BD 555359	X	
CD86	IT2.2	Nd	150	1.5	Fluidigm 3150020B		
IgM	MHM-88	Eu	151	1.2	Biolegend 314527	X	
CD11c	B-ly6	Sm	152	0.5	Biolegend 344102	X	
CD45RA	HI100	Eu	153	0.3	Biolegend 304102	X	

Antibody	Clone	Metal	Channel	Titer (Vol/rxn)	Manufacturer	CellCnn surface	CellCnn cytokine
CD14	M5E2	Sm	154	0.5	Biolegend 301802	X	
CD27	L128	Gd	155	1	Fluidigm 3155001B	X	
ICOS	C398.4A	Gd	156	1.5	Biolegend 313502		
IL-1RA	AS17	In	157	1.5	Santa Cruz sc-52775		X
Mip1-β	D21-1351	Gd	158	0.3	BD custom		X
PTEN	A2B1	Tb	159	1	BD 559600		X
IL-8	E8N1	Gd	160	0.5	Biolegend 511402		X
CD1c	L161	Dy	161	1	Biolegend 331502	X	
PD-1	EH12.2H7	Yb	162	1.5	Biolegend 329902	X	
CD19	SJ25C1	Dy	163	1	Biolegend 363002	X	
IL-6	MQ2-13A5	Dy	164	1	Biolegend 501102		X
CD16	B73.1	Ho	165	1	Fluidigm 3165007B	X	
TNF-α	MAb11	Er	166	0.7	Biolegend 502902		X
HLADR	M-A251	Er	167	0.5	Biolegend 356102	X	
CD56	REA196	Er	168	0.3	Miltenyi Biotech 130-108-016	X	
Il-1β	H1b-98	Nd	169	0.8	Biolegend Custom		X
MCP-1	5D3-F7	Er	170	1	eBioscience 14-7099-85		X
IL-12p40	C8.6	Yb	171	0.75	Biolegend 508804		X

Antibody	Clone	Metal	Channel	Titer (Vol/rxn)	Manufacturer	CellCnn surface	CellCnn cytokine
CD38	HIT2	Yb	172	1.5	Biolegend 303502	X	
CXCR5	RF8B2	Nd	173	2	BD 552032	X	
CD4	SK3	Yb	174	0.75	Fluidigm 3174004B	X	
IFN- α	LT27:295	Lu	175	0.5	Miltenyi Biotech 130-092-604		X
IL-23p19	23dcdp	Yb	176	0.75	eBioscience Custom		X
CD11b	ICRF44	Bi	209	0.75	Fluidigm 3209003B	X	
